# Molecular mechanism for bidirectional regulation of CD44 for lipid raft affiliation by palmitoylations and PIP2

**DOI:** 10.1371/journal.pcbi.1007777

**Published:** 2020-04-09

**Authors:** Fude Sun, Carsten F. E. Schroer, Carlos R. Palacios, Lida Xu, Shi-Zhong Luo, Siewert J. Marrink

**Affiliations:** 1 Beijing Key Laboratory of Bioprocess, College of Life Science and Technology, Beijing University of Chemical Technology, Beijing, China; 2 Key Laboratory of Molecular Biophysics, Hebei Province, Institute of Biophysics, School of Science, Hebei University of Technology, Tianjin, China; 3 Groningen Biomolecular Sciences and Biotechnology Institute, University of Groningen, Nijenborgh, Groningen, The Netherlands; Heidelberg Institute for Theoretical Studies (HITS gGmbH), GERMANY

## Abstract

The co-localization of Cluster-of-Differentiation-44 protein (CD44) and cytoplasmic adaptors in specific membrane environments is crucial for cell adhesion and migration. The process is controlled by two different pathways: On the one hand palmitoylation keeps CD44 in lipid raft domains and disables the linking to the cytoplasmic adaptor, whereas on the other hand, the presence of phosphatidylinositol-4,5-biphosphate (PIP2) lipids accelerates the formation of the CD44-adaptor complex. The molecular mechanism explaining how CD44 is migrating into and out of the lipid raft domains and its dependence on both palmitoylations and the presence of PIP2 remains, however, elusive. In this study, we performed extensive molecular dynamics simulations to study the raft affinity and translocation of CD44 in phase separated model membranes as well as more realistic plasma membrane environments. We observe a delicate balance between the influence of the palmitoylations and the presence of PIP2 lipids: whereas the palmitoylations of CD44 increases the affinity for raft domains, PIP2 lipids have the opposite effect. Additionally, we studied the association between CD44 and the membrane adaptor FERM in dependence of these factors. We find that the presence of PIP2 lipids allows CD44 and FERM to associate in an experimentally observed binding mode whereas the highly palmitoylated species shows no binding affinity. Together, our results shed light on the sophisticated mechanism on how membrane translocation and peripheral protein association can be controlled by both protein modifications and membrane composition.

## Author summary

Cytoskeleton protein complex involving with association of CD44 and ERMs is critical for cancer-related cellular adhesion and migration. The protein interactions are found to be modulated by chemical modification and membrane microenvironments, but the inherent mechanism is unclear. We obtained molecular dynamic details of CD44 localization switching between raft/non-raft subdomains regulated by palmitoylations and PIP2 molecules. Binding of PIP2 on the palmitoylated CD44 enables it to release from lipid raft, revealing an exceptional role of PIP2 in mediating protein translocation. PIP2 is beneficial for CD44 to associate with the active domain of ERM, in a nearly crystal structure mode. The molecular information will enhance our understanding for PIP2 regulation to protein translocation and membrane association.

## Introduction

The Cluster-of-Differentiation-44 protein (CD44) is a versatile molecule that is involved in a variety of cellular processes, including inflammation, hematopoiesis, cell migration and cancer invasiveness [1–3]. The protein consists of an ectodomain (ED), a single transmembrane domain (TMD), and a cytoplasmic tail (CT). The ED can undergo different modifications, like hyaluronic acid binding and proteolytic shedding, which are regulated by the localization of the TMD and possible self-assembly. In a similar way, the localization of CD44 into the ordered, “lipid raft” [4,5], microdomains can suppress the binding of the CT to cytoskeleton adaptors of the Ezrin, Radixin and Moesin (ERM) family, which act as regulators of cell motility and differentiation [6,7]. The translocation of CD44 from non-raft into raft domains is driven by palmitoylations on the cysteine residues [8]. Moreover, the palmitoylations on CD44 control the association/dissociation switching of CD44 and Radixin, a process, that has been identified in tumor cell migration and proliferation [9].

In addition to protein modifications, specific lipids play a role in regulating the behavior of CD44. In particular, the signaling lipid phosphatidylinositol 4, 5-bisphosphate (PIP2) has drawn extensive attention because of its special function during protein clustering and translocation [10,11]. PIP2 is a multivalent anionic lipid that exclusively resides in the inner leaflet of the cell membrane with a molar fraction of 2–5% [12,13]. In model membranes, PIP2 resides in the disordered (non-raft) domain, presumably due to the high unsaturation on the sn-2 hydrocarbon tail [14]. In living cells, however, the existence of distinct microdomains is still under discussion, and the heterogeneous distribution of PIP2 appears restricted to the nanoscale [15,16]. Regardless of the exact location of PIP2 in the membrane, the association between CD44 and Ezrin is accelerated in the presence of PIP2 [17]. In addition, it has been found that the activation of the N-terminal domain of ERM protein (FERM), which is responsible for the membrane binding of the ERM, is induced by PIP2-binding [18], and the binding of FERM to membranes is strongly stabilized by even low concentrations of PIP2 [19]. These results suggest that PIP2 facilitates the association of CD44 and ERM by activating the FERM protein, supporting the association of FERM to the membrane surface and translocating both to the non-raft domain.

A comparison between the effect of palmitoylations and PIP2 lipids on the activity of CD44 reveals that both factors have opposite effects. Studies, that are exploring the balance between protein acetylations on the one hand and lipid composition on the other hand are however lacking so far. Despite the biotechnological advances in recent years, there is still some difficulty in obtaining experimental insights into protein distribution and translocation on a molecular level. Molecular dynamics (MD) simulations, especially based on the Martini coarse-grained (CG) force field, have become a powerful alternative to study the interaction of membrane components in detail [20–23]. In the Martini CG force field, groups of four non-hydrogen atoms are represented by a CG bead, resulting in an expansion of the available simulation time scales and system sizes while offering a near-to-atomistic resolution. The Martini model has been successfully applied to a variety of membrane related processes, including the bilayer segregation into ordered and disordered domains [24,25], membrane protein-protein assembly [26–28], protein-lipid interplay [29–31], and protein sorting between domains [32–34]. Furthermore, plasma membrane models exist within the framework of the Martini force field that allows one to study the properties of lipids bilayers in a biologically relevant context [35,36].

In this CG MD study, we analyze the properties of CD44 in a phase–segregated model membrane with respect to its membrane distribution and FERM affinity, in dependence of the palmitoylation degree and the presence of PIP2. These results are tested against a more realistic plasma membrane model. We detail the effect of both factors on the localization and conformation of the peptide as well as on the implications on formation of the CD44-FERM complex. These insights can help to obtain a broader understanding on the balance between these factors and how they can be influenced in a cellular context.

## Results

### Palmitoylation Increases CD44 Raft Affiliation

**Fig 1:**
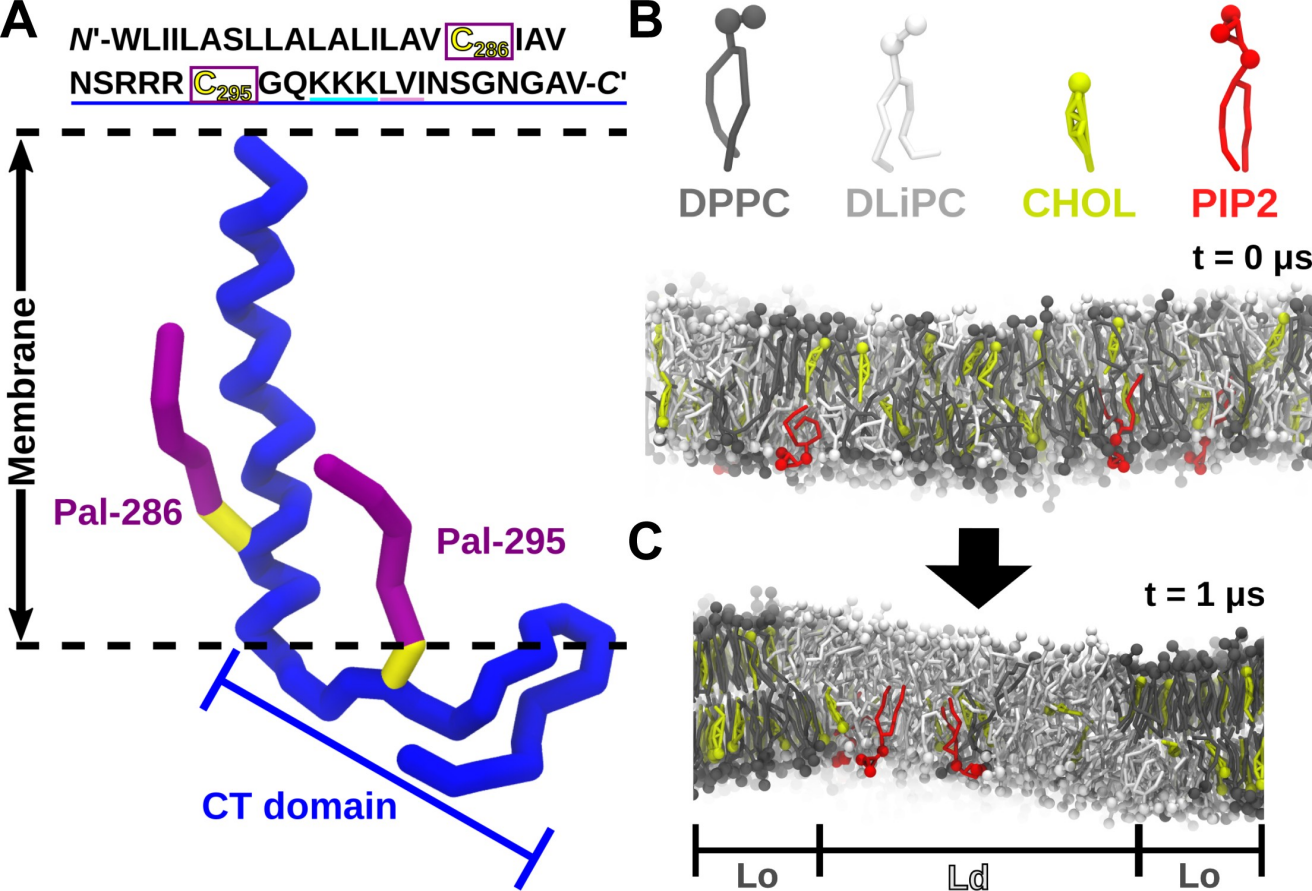
System setup. (A) CD44 Sequence and structure of the double palmitoylated CD44 in the CG representation. Only the backbone beads of CD44 are shown. The palmitoyl moieties are shown by purple chains, connected to the cysteine 286 and 295 (shown in yellow) respectively. (B) CG lipid models of DPPC, DLiPC, CHOL and PIP2. (C) Evolution from the initially randomly mixed membrane into coexisting phases of liquid-disordered (*Ld*) and liquid-ordered (*Lo*) subdomains.

**Fig 2:**
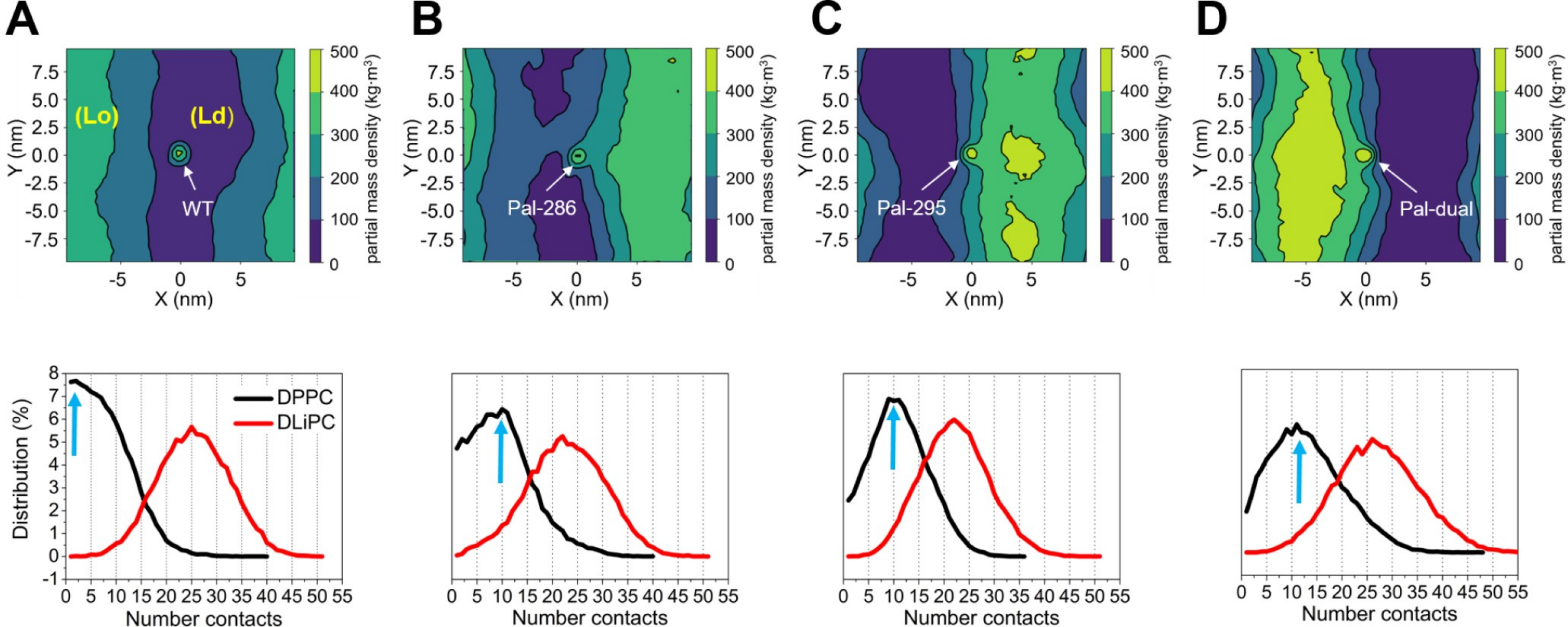
Sorting of CD44 toward raft domains upon palmitoylation. The upper panels show the 2D density maps of the positions of the (A) CD44-WT, (B) Pal-286, (C) Pal-295 and (D) Pal-dual relative to the *Lo* phase. Only the density of DPPC is taken into consideration to determine the position of the raft domain (*Lo*). The lower panels show the probability distribution of contacts between DPPC (black) or DLiPC (red) and (A) CD44-WT, (B) Pal-286, (C) Pal-295, and (D) Pal-dual, respectively. The arrows indicate the most likely DPPC contacts for each CD44 variant (2 in case of CD44-WT, 10 for Pal-286 and Pal-295 and 12 for Pal-dual). The numerical data used in the lower panels can be found in S1 Data.

To reveal the preferred partitioning of CD44 between raft and non-raft membrane domains, we simulated a membrane composed of 40%dipalmitoyl-phosphatidylcholine (DPPC)/40%dilinoleoyl-phosphatidylcholine (DLiPC)/20%cholesterol (CHOL) which rapidly segregates into two subdomains (Fig 1C), as observed previously [25]. One of the domains, the liquid-ordered (*Lo*) domain or "raft" domain, is enriched in DPPC and cholesterol, whereas the other domain, the liquid-disordered domain (*Ld*), is enriched in DLiPC. Simulations including a single copy of the wild-type (WT) of CD44 in the membrane reveal that CD44-WT prefers to reside in the DLiPC-enriched *Ld* phase, as apparent from a snapshot at the end of the simulation as well as analysis of the contact frequency distribution of the protein with the two lipid types (Fig 2A). The clear location preference of CD44-WT in the non-raft domain is consistent with experimental observations [9]. A simulation with a duration of 6.0 μs is used for analysis. Within the first microsecond, we observe a separation of the bilayer in two phases where CD44 has a clear tendency reside in the DLiPC enriched *Ld* phase, indicated by a larger number of contacts to DLiPC (see S1 Fig). After the phase separation occurred, the number of contacts is not static but fluctuates, thereby indicating a dynamics local environment.

To probe the effect of the palmitoylation on the localization preference of CD44, the simulations were repeated with two single palmitoylated CD44 proteins (Pal-286 and Pal-295) as well as with a double palmitoylated CD44 (Pal-Dual, Fig 1A). In contrast to the CD44-WT, both Pal-286 and Pal-295 prefer to reside at the boundary of the raft domain (Fig 2B and 2C). The amount of DPPC-contacts of the single palmitoylated CD44 increases (as highlighted by the arrows) for both Pal-286 and Pal-295. CD44-Pal-dual exhibits an even stronger effect compared to the single palmitoylated species, as the peak of the most frequent contact shifts to more contacts and the profile shape broadens towards a higher contact range (Fig 2D). Although the palmitoylated CD44s are not found to entirely enter the *Lo* domain, it is evident that an enhanced degree of palmitoylation increases the raft affinity of CD44 (S2 Fig). A previous study on WALP proteins shows that the attachment of two palmitoyl anchors on WALP23 is not sufficient to translocate the proteins into the *Lo* phase and that only an addition of eight palmitoyl anchors allows the protein to move into the raft domain of strongly phase separating ternary model membranes [32]. Additionally, it was recently demonstrated that the affinity of TM proteins to the phase boundary is somewhat higher than to the lipid raft itself and only a saturation of the phase boundary with several TM proteins allows them to enter the raft domain [34]. This may be the reason why we cannot observe a full translocation into the raft domain here, although the addition of palmitoylations clearly increases the raft affinity of CD44.

**Fig 3:**
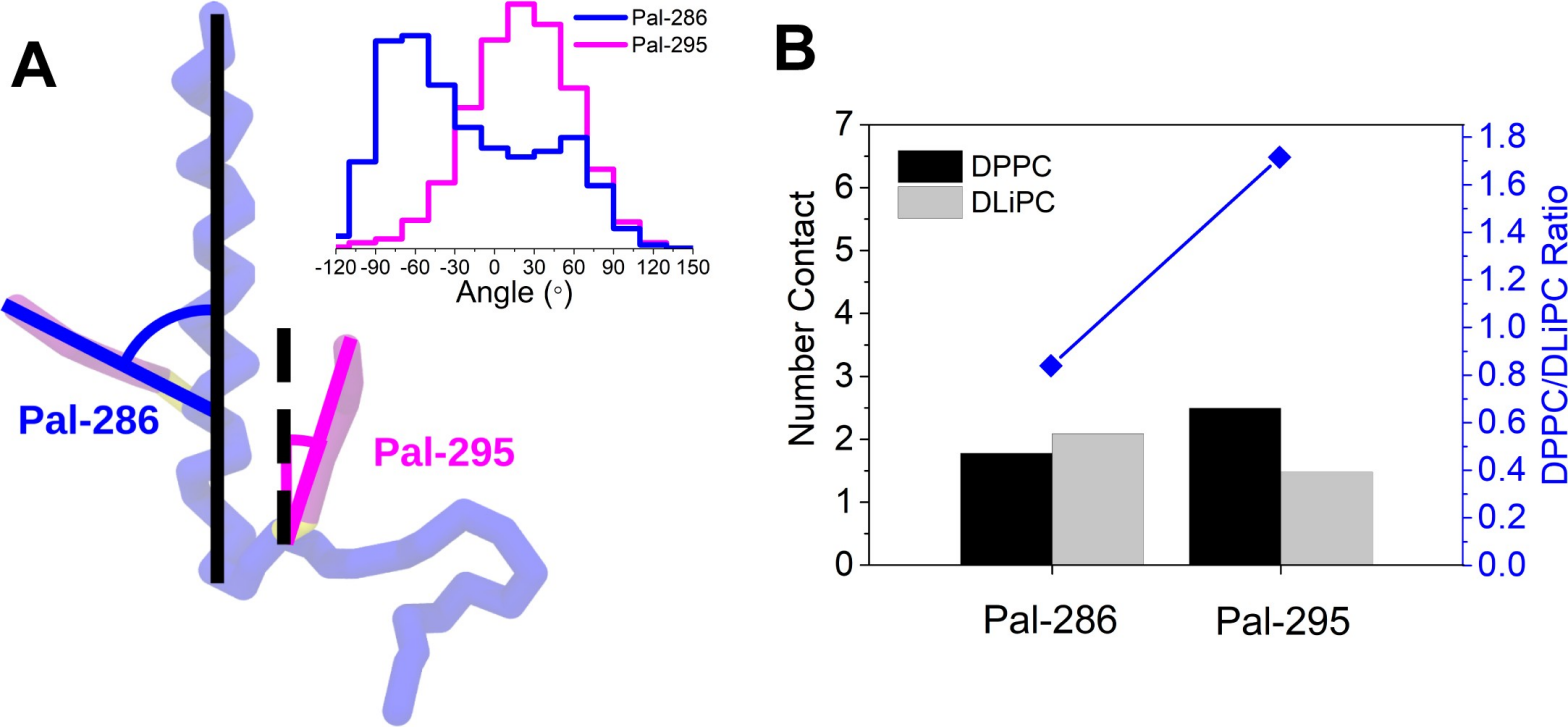
Orientation of double palmitoylated CD44 at raft boundary. (A) A snapshot shows the respective orientation of the two palmitoyl moieties, relative to the membrane normal. The respective angle distributions are shown in the upper-right corner. The numerical data used in the upper-right panels can be found in S1 Data. (B) Average number of lipid contacts from Palmitoyl-286 and Palmitoyl-295, respectively. The ratio of DPPC/DLiPC contacts are shown in the upper panel.

For both the single palmitoylated CD44 proteins, the orientation at the raft boundary is such that the palmitoyl chain is oriented toward the raft domain. This is expected given the fully saturated nature of the anchor. For the double palmitoylated chain, however, there are multiple orientations possible as the palmitoylations are on different sides of the protein. When inspecting the orientation of the two palmitoyl anchors on the Pal-dual relative to the membrane normal (Fig 3A upper-right), the palmitoyl-chain at Cys286 is found to adopt an unfavorable angle perpendicular to the membranes normal and, thus, to the lipid tails. In contrast, the palmitoyl chain at Cys295 inserts parallel into the bilayer. To further quantify the different affinities of both palmitoylation sites, we computed the average number contact of both DPPC and DLiPC (Fig 3B). It can be seen that the palmitoyl chain at Cys-286 shows nearly the same affinity for both DPPC and DLiPC (ratio DPPC/DLiPC: 0.85), whereas the palmitoyl chain at Cys-295 shows a clear preference for DPPC (ratio DPPC/DLiPC: 1.7). This result indicates that the palmitoylation site Cys-295, located at the C-terminus of CD44 is more efficient in binding DPPC lipids than the Cys-286 site of the protein, and determines the orientation of CD44 with the Cys-295 anchor facing toward the raft domain.

### The Raft Affinity of CD44 is Reduced in PIP2 Containing Membranes

**Fig 4:**
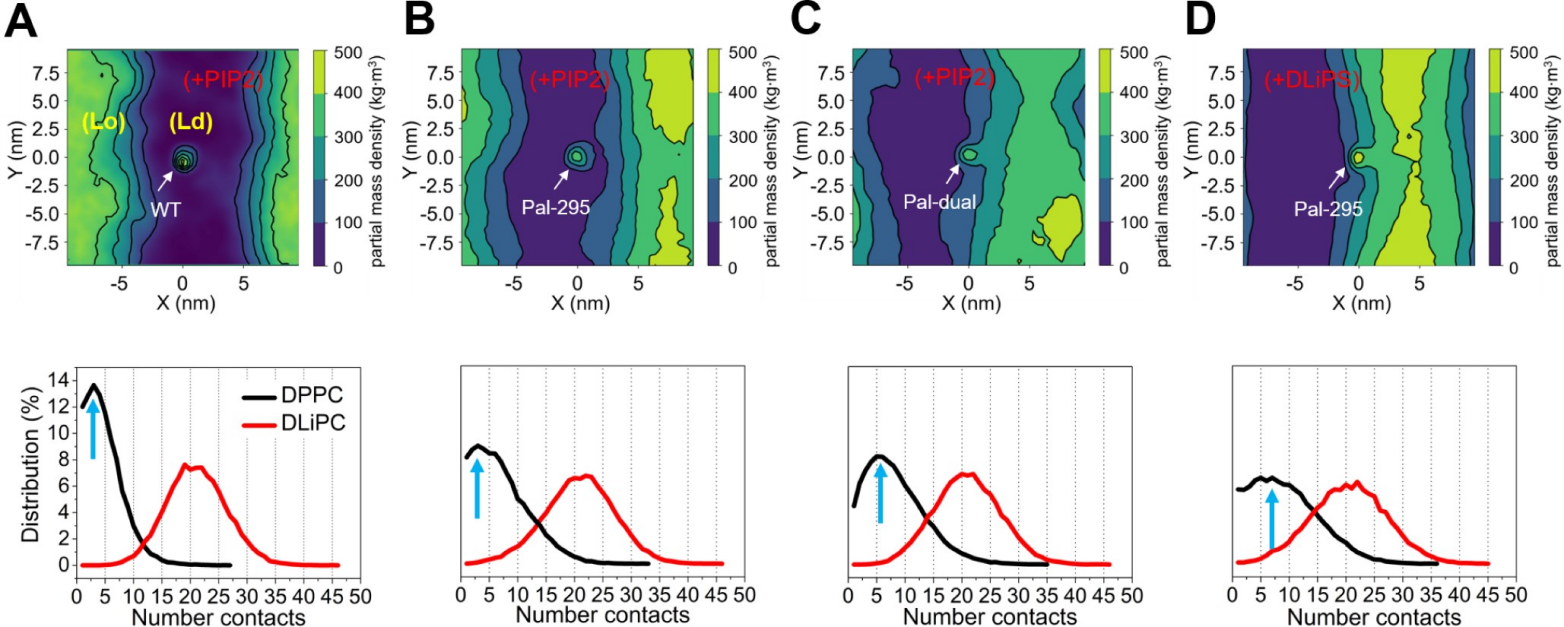
Effect of PIP2 on sorting of CD44. The upper panels show the respective locations of (A) CD44-WT, (B) the Pal-295, (C) the Pal-dual in the bilayer containing PIP2 lipids, and (D) the Pal-295 in the bilayer with DLiPS inclusion. The bottom panels correspondingly show the distribution of DPPC number contacts of the CD44 variants in different membrane environments. The numerical data used in the lower panels can be found in S1 Data.

Next, PIP2 was incorporated in the lower leaflet of our simulated membrane with a mole percentage of 2%. Consistent with fluorescence labeling tests on the giant unilamellar vesicles [14], PIP2 molecules prefer to distribute in the non-raft domain (Fig 1C). To investigate the influence of PIP2 on the location preference of CD44, we repeated the simulations of the palmitoylated variants with the PIP2-contained membrane. The CD44-WT maintains a preference to residing in the non-raft domain (Fig 4A). We thereby restricted the analysis of the single palmitoylated variants on Pal-295 due to its higher affinity to the Lo phase in absence of PIP2. The resulting frequency distribution of DPPC contacts of Pal-295 (Fig 4B) is similar to that of the CD44-WT, and quite different compared to the contact distribution of Pal-295 in a membrane without PIP2 (Fig 2C). This indicates that the presence of PIP2 lipids has the opposite effect on the sorting behavior of CD44 than the palmitoylations. However, a raft-affinity abolishment cannot be observed for the Pal-dual variant, which still remains at the raft boundary (Fig 4C). A second palmitoylation is thus able to outcompete the effect of the PIP2 lipids, allowing for a sophisticated regulation mechanism of the protein positioning.

**Fig 5:**
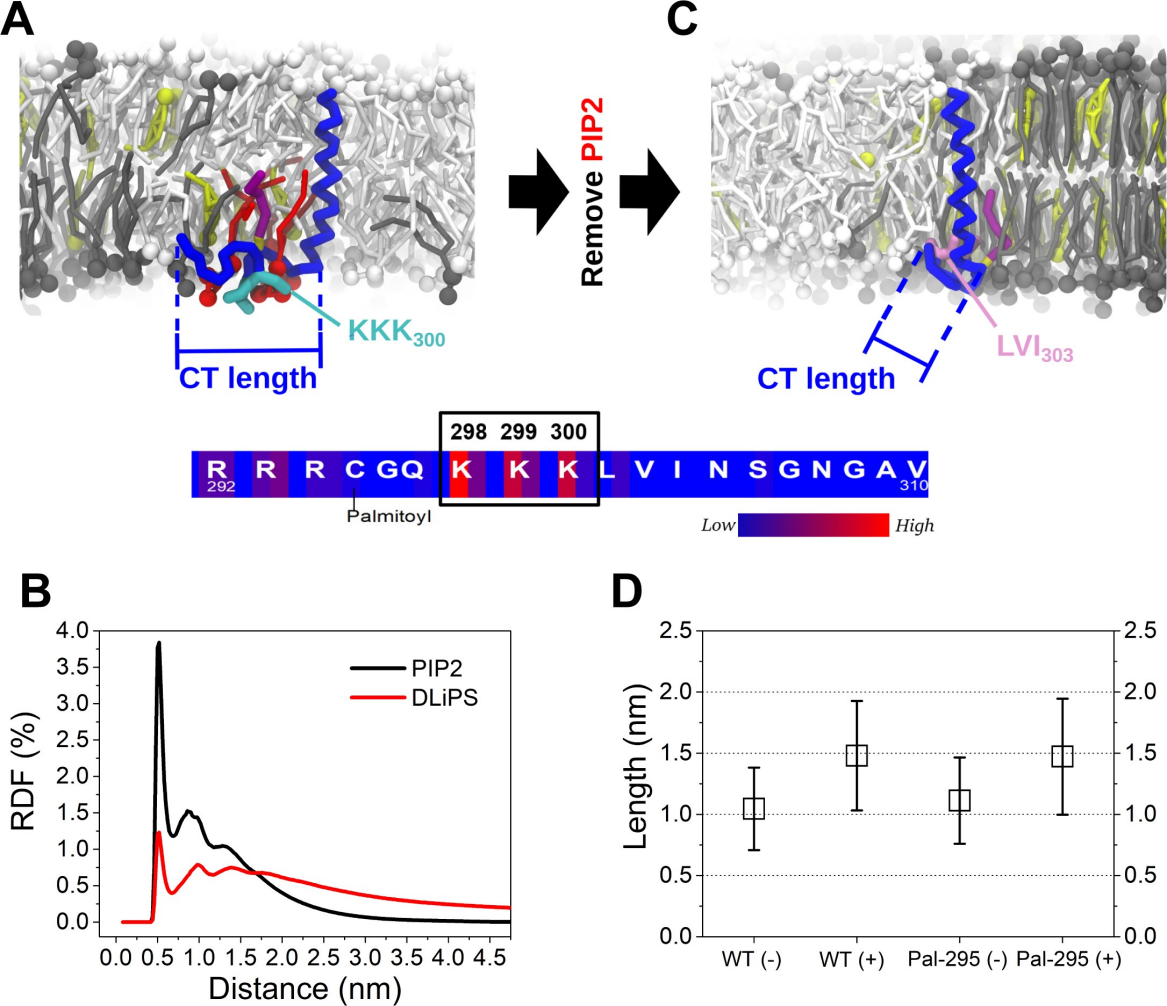
Specifics of PIP2 in CD44 binding. (A) A snapshot of the CD44-Pal-295 in binding PIP2 molecules and the frequency distribution of the involved residues binding the CT domain. The side chains of KKK_300_ are shown in cyan. (B) A comparison of radial distribution function (RDF) of PIP2 and DLiPS relative to CD44, respectively. (C) Presentation of the CD44-Pal-295 conformation in the bilayer after the removal of the PIP2 lipids. The LVI_303_ motive is shown in light purple. (D) The lengths of the CT domains of the CD44-WT and Pal-295 in the membranes including PIP2(+) and excluding PIP2(-), respectively. The length of the CT was determined by calculating the distance between the R_292_ and the V_310_ residues on the CT domain (indicated in (A). The bars denote standard deviations. The numerical data used in Fig 5A, 5C and 5D can be found in S1 Data.

To study the specifics of the regulatory mechanism of the PIP2 lipids, PIP2 in the system was replaced by DLiPS that has two polyunsaturated tails. DLiPS also has a high affinity for the *Ld* domain, but carries a single negative charge instead. The amount of DLiPS was adjusted to 10 mol% such that the charge density in the lower leaflet remained the same to PIP2 membrane. It can be seen, that Pal-295 cannot detach from the raft boundary under this condition, and the frequency distribution of DPPC contacts is shifted to a higher number of contacts (Fig 4D). Accordingly, PIP2 acts specifically as a *Ld* driving factor. As a hint on what causes the specific driving force, we observe a pronounced clustering of PIP2 around CD44, which cannot be detected for DLiPS (Fig 5A and 5B). The reason for this owes to the highly basic KKK_300_ motif close to the palmitoylation site at Cys295 that can accumulate the multivalent PIP2 lipids (lower scheme of Fig 5A). Due to the affinity of PIP2 to the non-raft domain, Pal-295 is eventually translocated in the non-raft domain, thus disabling the effect of the palmitoylation (Fig 5A). In a previous study we found that PIP2 has a strong tendency to cluster around the juxtamembrane domain of L-selectin and facilitates its association with the FERM adaptor [37]. In accordance to the present results, the electrostatic interaction between the PIP2 head groups and the basic residues in the CT of the molecule could be identified to drive the clustering of PIP2 lipids. In contrast, monovalent lipids, that also retain *Ld* preference, were not able to undergo the pronounced clustering. Our results thus provide strong evidence that the interplay between basic residues close to the membrane surface and the highly charged PIP2 lipids is an important pathway for controlling signaling proteins like L-selectin and CD44.

PIP2 is furthermore found to regulate the conformation of the CT domain of CD44. In the membrane without PIP2, the CT domain is partially folded since this allows the hydrophobic motif LVI_303_ (next to the KKK_300_) to access the interior of the membrane (Fig 5C). In contrast, the presence of PIP2 enables the CT domains of both WT and Pal-295 to exhibit a more extended conformation (Fig 5D). Apparently, the strong interaction between the charged head groups of PIP2 and the KKK_300_ motif of CD44 reduces the conformational freedom of the hydrophobic region. The PIP2 induced conformational change of the CT is likely to have an influence on the ability of CT to bind to membrane adaptors such as FERM, which we address next.

**Fig 6:**
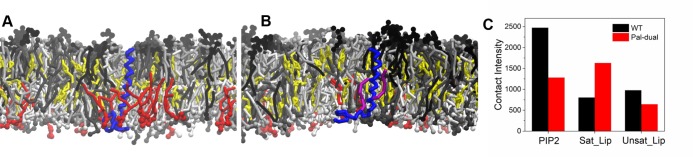
CD44 in a plasma membrane environment. (A), (B) Snapshots of CD44-WT (A) and CD44-Pal-dual in the plasma membrane. The color code of CD44, PIP2 and cholesterol is the same as in Fig 1. The other lipids are colored in shades of gray according to their degree of saturation: fully saturated lipids are depicted in black, single and double unsaturated lipids are shown in dark and light gray, and highly unsaturated lipids are colored in white. More details can be seen in the Methods part. (C) Contact intensity of the CD44 variants with PIP2, the fully saturated and the highly unsaturated lipids. The numerical data used Fig 6C can be found in S1 Data. More definition details can be seen in the Methods part.

To check whether the results obtained for the ternary system hold up in a more complex environment, we conducted additional simulations of CD44-WT and CD44-Pal-dual in a model plasma membrane. The plasma membrane thereby consists of 19 different types of lipids (including PIP2 and cholesterol) with various degrees of saturation that are asymmetrically distributed in the upper and lower leaflet (exact lipid composition can be found in Methods section). Simulation snapshots of the system are depicted in Fig 6A with CD44-WT and Fig 6B with CD44-Pal-dual. It can be observed that, in contrast to the ternary system, there is not a well defined phase boundary; the distribution of the lipids is much more homogeneous. In the vicinity of the protein however, lipid sorting can be observed, e. g. the high abundance of PIP2 (shown in red) around CD44-WT in Fig 6A.

To quantify this behavior, we computed the contact intensity of both CD44 variants with PIP2, the saturated lipids and the highly unsaturated lipids. In Fig 6C, the results of this analysis are displayed. It can be seen that CD44-WT has indeed a stronger an affinity to PIP2 as compared to the palmitoylated protein. Furthermore, the environment of the wild type contains significantly more highly unsaturated lipids which are thus co-localized around CD44-WT. In contrast, the palmitoylated protein sequesters more saturated lipids and displays a lower affinity for PIP2. This behavior is in full agreement with the ternary system: the wild-type of CD44 prefers an environment rich in PIP2 and unsaturated lipids (similar to the *Ld* phase), whereas the palmitoylations lead to an accumulation of saturated lipids around CD44 (similar to the *Lo* phase).

### Association of CD44 to FERM is Regulated by PIP2

One of the important functions of CD44 is to associate with the cytoskeletal adaptor FERM which may be influenced by the presence of PIP2 lipids in various ways. First, recent experimental [19] and simulation data [37] show that the presence of PIP2 strongly facilitates the binding of FERM to the membrane surface. Second, there is evidence that FERM is mainly active in the non-raft domain whereas, as we have shown above, the localization of CD44 is dependent on its palmitoylation state and the presence of PIP2. Third, PIP2 addition is able to induce a structural extension of the CD44-CT domain that can further affect the protein association of CD44 and FERM. To investigate this mechanism in more detail, we created a CG model of the FERM domain of Radixin and studied its association to CD44-WT in presence of PIP2.

**Fig 7:**
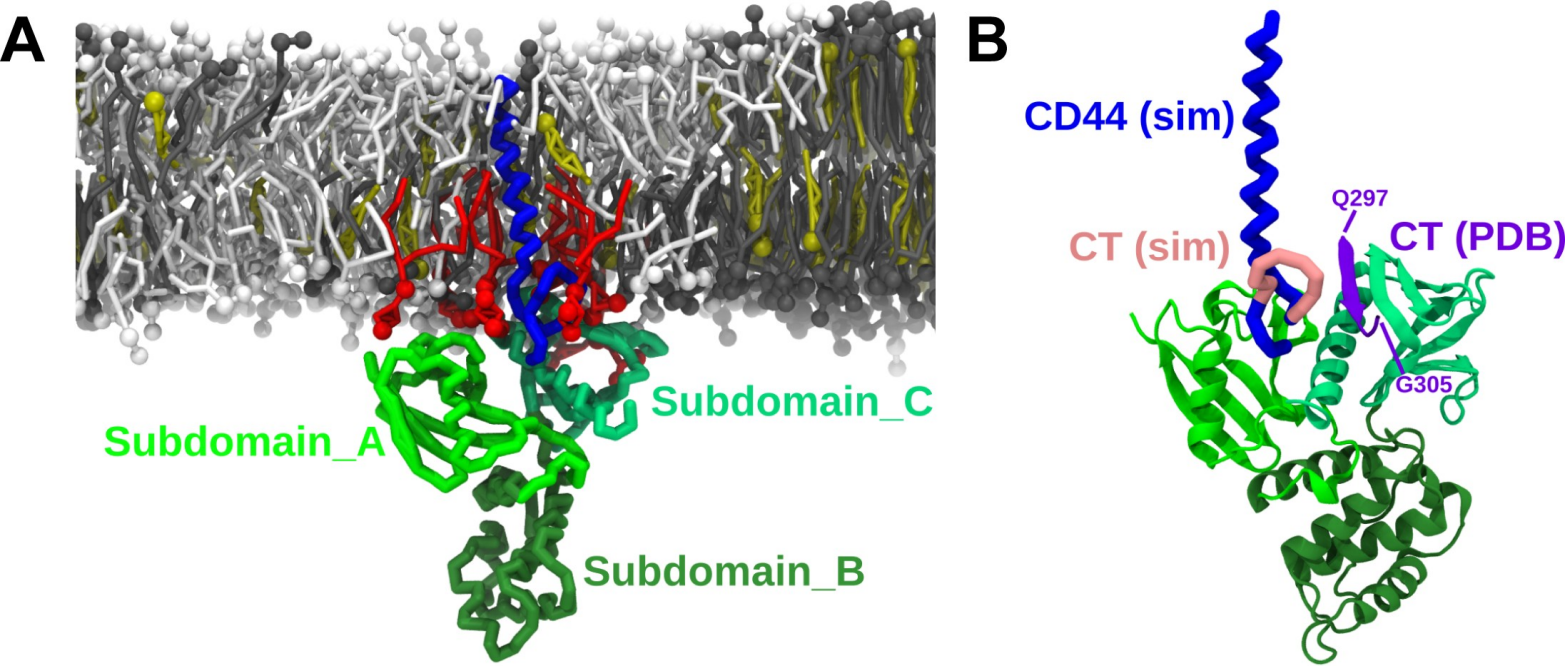
PIP2-dependent formation of CD44-FERM complex. (A) Presentation of CD44-FERM conformation and association on the membrane surface in the presence of PIP2. The backbone structure of CD44 is shown in blue, the FERM domain is shown in different shades of yellow/green, dependent on the subdomain. The snapshot in a bottom-view is exhibited in the left-upper corner. B) Comparison of the binding mode from our simulation with the crystal structure of the FERM-CT complex. The FERM domain of both structures are aligned. The CT domain (Q297-G305) in the crystal structure is colored in purple, and in pink in our simulation.

Our simulation was set up in such a way, that the FERM domain was positioned at a distance of 1.0 nm away from the lower leaflet of the membrane, and laterally separated from CD44 by 5 nm. We observe that FERM attaches to the *Ld* of the membrane via the PIP2 lipids. FERM is thereby oriented in such a way that the subdomains A and C are in direct contact with the membrane (Fig 7A, S3A Fig). The same contact mode of FERM with the membrane can also be observed when the initial distance is increased to 2 nm, as well as in a simulation starting from a bottom-up rotation of FERM (S4 Fig). CD44, which is co-localized in the *Ld* domain, approaches FERM and forms a protein complex that remains stable for the remaining 3 μs of the simulation (Fig 7A, S3C and S3D Fig). This complex is surrounded by a cluster of PIP2 lipids which is in agreement of what we have observed for the association of FERM and L-selectin [37]. Furthermore, its localization in the *Ld* domain is line with experimental insights [8,9]. The localization preference of protein complex for the *Ld* derives from the phase preference of the PIP2 lipids that are mediating the membrane attachment (S5 Fig). Within the protein complex, CD44 resides in the middle of the two subdomains A and C which agrees with the crystal structure of the CT of CD44 bound to FERM [6]. The same stable protein binding mode is observed in a second simulation replica (S6 Fig).

For a more detailed comparison of the binding mode between the two proteins, we took the protein structure from the Fig 7A and the crystal structure from (pdb code: 2zpy) [6] then aligned the FERM domains of the protein complex. The alignment result is shown in Fig 7B. Due to the CG nature of the simulation, the beta-sheet-like attachment of the CT to FERM cannot be reproduced, however, the CT of CD44 is located very close to its counterpart in the crystal structure and displays the same alignment relative to FERM (more association detail is seen S6 Fig). The CT resides next to the binding site between helix α1C and strand β5C of the subdomain_C but the fragment Q297-G305 (colored in pink) bends up towards the membrane. A reason for this behavior may be due to the fact, that the PIP2 enrichment in vicinity of CD44 is weakened when FERM is at present (S7 Fig). This can lead as we discussed above (Fig 5C) to a folding of CD44-CT. Nevertheless, as the binding geometry was not enforced in our simulation by any means, this behavior indicates a clear driving force for the proteins to associate in this particular arrangement.

**Fig 8:**
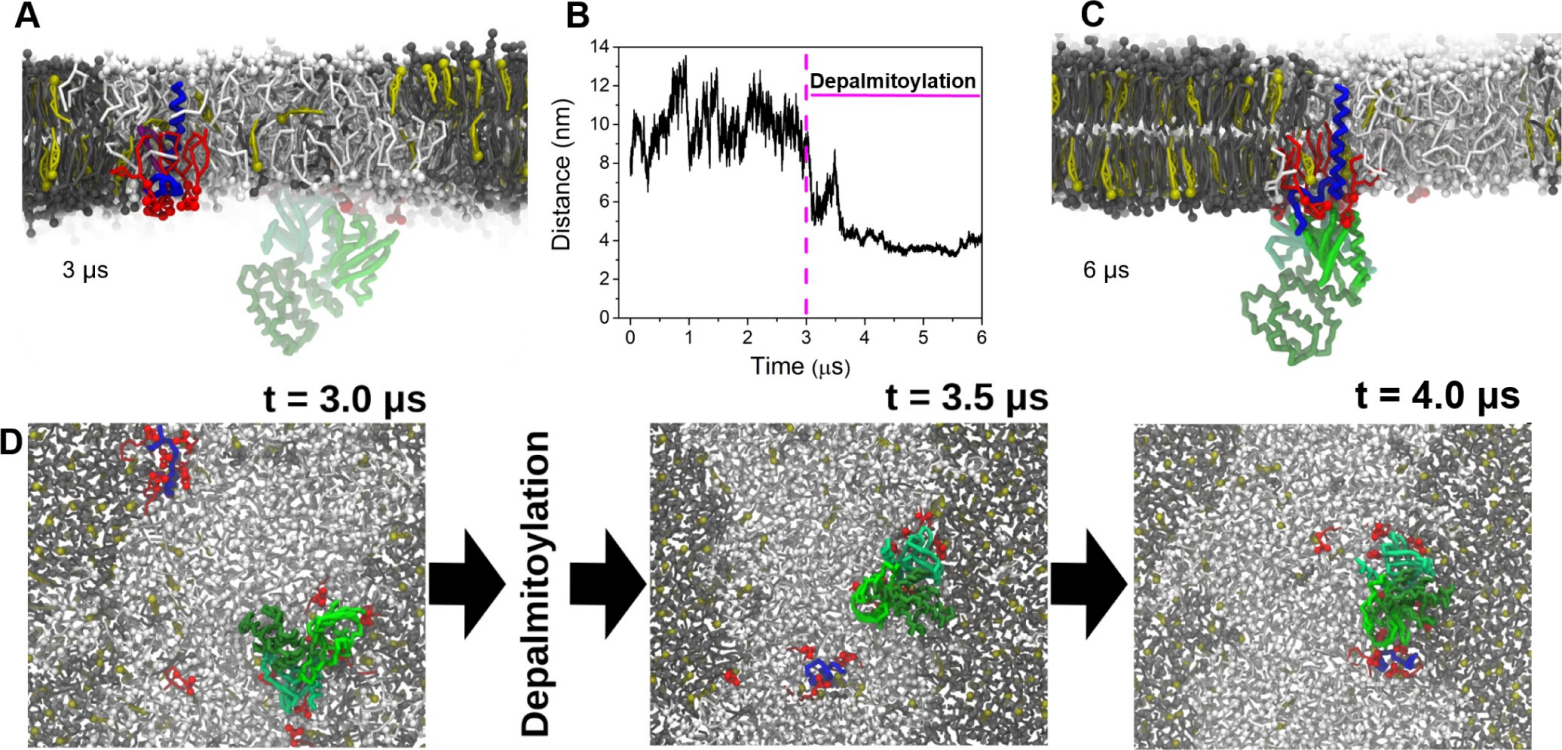
Anti-regulation of palmitoyl anchors and PIP2 lipids. (A) A snapshot presenting the separated state of CD44-Pal-Dual and the FERM domain. CD44 is locked on the phase boundary with the palmitoyl anchors orientating to the raft domain. (B) Distance evolution of CD44 and FERM within 0–3.0 μs (CD44-Pal-Dual) and 3.0–6.0 μs (CD44-depalmitoylation). The numerical data used in Fig 8B can be found in S1 Data. (C) Binding state of CD44-FERM at the final simulation time in the depalmitoylated case of CD44. (D) Protein-protein association processes, shown from the perspective of the cytosol. The left panel shows the proteins right before the depalmitoylation, the central panel the state 500 ns after this event, the right panel the structure after protein association.

In contrast to the spontaneous binding of CD44-WT to FERM, the Pal-Dual and FERM do not show any binding whatsoever during a 6.0 μs simulation (either in two time-extending simulations, S8 Fig). FERM binds to the membrane surface, a process mediated by PIP2 lipids as we showed in our previous work [37], but is unable to complex CD44. The lack of hetero complex formation is easily rationalized by their different locations at the phase boundary (Pal-Dual) versus the non-raft domain (FERM), as shown in Fig 8A. Upon removal of the palmitoylation groups of Pal-Dual, CD44 no longer resides at the raft domain boundary and eventually associates with FERM in the non-raft domain (Fig 8B and 8C and S8 Fig). The results thus highlight the competition of palmitoylation and PIP2 in association of CD44 and FERM. Furthermore, the formation of the CD44-FERM complex is driven by a merging of the PIP2 clusters occupying the annular shells of both proteins (Fig 8D). The results are consistent with our previous findings on complex formation between FERM and L-selectin, revealing a positive regulation of PIP2 lipids in protein-protein interactions in membranes. Note that for all simulations, if not stated differently, the lipids in the bilayer are initially randomly distributed (as shown in the upper panel of Fig 1C). As the lipid distribution at the beginning of the simulation is randomized, the environment of CD44 and FERM is not biased, the attachment of FERM via PIP2, the clustering of PIP2 around CD44 and the subsequent cluster merging are therefore a result of their molecular driving forces rather than the simulation setup.

It was noteworthy that the cytoplasmic domain of CD44 is not loosely stretching into the cytosolic solution, making it difficult for the FERM to directly bind to CD44. Therefore, the membrane-attachment of the FERM domain via PIP2 lipids acts as the first step for the formation of protein hetero complex. After adsorbing to the membrane, FERM is still mobile and able to diffuse within the boundaries of the *Ld* phase (S9 Fig). Taken together, the results indicate the important role of PIP2 in guiding protein translocation and association, as an anti-regulation to palmitoylation modification.

## Discussion

The translocation of cell adhesion molecules inside cell membranes acts as a crucial step in connecting various cellular activities. Different composition in membrane microenvironments and specific modifications on the TM proteins are thought to determine the locational preference of the molecule, possible self-assembly as well as the formation of cytoskeletal actin networks. In this study, extensive molecular dynamics simulations are used to explore the underlying mechanism of CD44 translocation, mediated by palmitoylations and the presence of PIP2 lipids in the membrane. Our results unravel a delicate balance of protein moieties and membrane composition that strongly influences both the distribution of CD44 and its ability to bind the cytoskeletal adaptor FERM.

Lipid rafts are known to act as organization center for various transmembrane proteins such as amyloid precursor proteins. In breast cancer tumor cells, the localization of CD44 in lipid rafts, induced by palmitoylations on its cysteine residues, is considered to avoid the migration of these cells. A removal of the moieties, e.g. by site directed mutagenesis, transfers CD44 into the non-raft domains which is in agreement with the organization in invasive cancer cells. Moreover, palmitoylations are also found to be important for CD44-hyaluron acid (HA) turnover [38], what is in line with the observation that lipid rafts, which can bind CD44 and thus traffic HA, are promoting endocytosis [39].

In the context of the discussion on how CD44 is acting in the development of cancer, the translocation of the protein into and out of the lipid raft domain appears to be a crucial factor. In our simulations we find, in line with experimental results, that CD44-WT prefers to reside in the non-raft domain, while attachment of palmitoyl groups increases the raft affinity. In our plasma membrane model, that does not display a phase separation but instead shows a lateral organization characterized by transient nano domains, we observed a similar trend: CD44-WT accumulating PIP2 and unsaturated lipids in its vicinity, while the introduction of palmitoylations enhances the ability of CD44 to sequester more saturated lipids. Co-localization of CD44 and the cytoskeleton adaptor is found to promote in invasive cancer cells [9,40] and it is speculated that the ERMs sequester CD44 in the non-raft domain to accelerate the cellular migration. It appears therefore reasonable that palmitoylations can suppress the spread of tumor cells by keeping CD44 and the ERM adaptors in separate micro- or nano-domains of the membrane.

**Fig 9:**
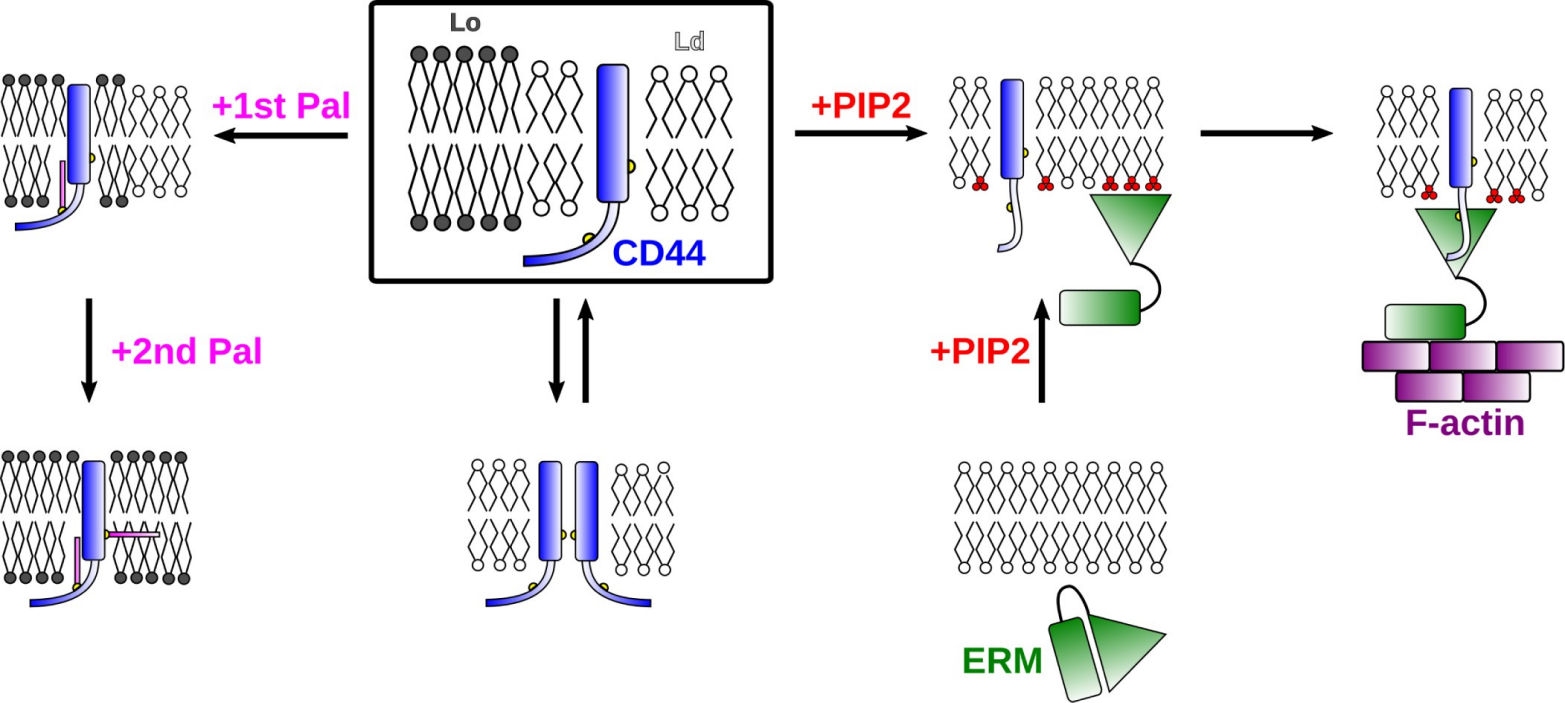
Schematic representation for the membrane translocation and protein association of CD44 regulated by palmitoylations (left branch) and PIP2 (right branch) in the different micro- or nano-domains.

However, the magnitude of the effect of the palmitoylations on the translocation of CD44 appears to be dependent on both their number and their position [8,41]. The palmitoylation site at Cys-286 is located in the TM and thus buried in the membrane, whereas the Cys-295 is located at the disordered CT. Cys-295 is likely to be easier palmitoylated by palmitoyl acetyltransferases (PATs) that reside in the raft domain which, as we show here, cannot be readily entered by the TM of CD44 without palmitoyl moieties. Furthermore, it is known that CD44 can form homodimers or dimers with other growth factors in which Cys-286 is involved in the intermolecular contact [42,43]. This contact can act as another barrier for the palmitoylation of the TM site (see Fig 9 and S10 Fig). Our results could show that a palmitoylation at Cys-295 can enhance the raft affinity of CD44 and is capable to sequester more raft lipids than Cys-286 due to a preferred binding geometry with the lipid. A previous study shows that the dimerization state of TM proteins can be interrupted in presence of the lipid raft [44]. This suggests a mechanism, in which CD44 is palmitoylated at Cys-295 to migrate into the raft domain, where possible dimers are dissociated and a second palmitoylation at Cys286 through PAT can take place, reinforcing the phase preferences of the protein (left branch in Fig 9)

The association of CD44 and the FERM domain can be accelerated in the presence of multivalent PIP2 lipid (right branch in Fig 9). Thereby, three mechanisms in which PIP2 is acting on CD44 and FERM can be identified: First, the presence of PIP2 lipids allows FERM to bind to the surface of the membrane. PIP2 allows the full ERM to transfer into its active form [18] and subsequently enhances the binding affinity of the FERM to the membrane [19,45,46]. Since PIP2 prefers to reside in the non-raft domain due to its unsaturated bonds in *sn*-2 hydrocarbon tail, FERM will be localized at the non-raft domain as well. Second, PIP2 lipids enable single palmitoylated CD44 to migrate to the non-raft domain, thus allowing CD44 and FERM to be co-localized. The change of the raft affinity is thereby achieved by a clustering of PIP2 around the basic residues at the surface between CT and TM that effectively shield the moieties from interacting with the raft lipids. Also for CD44-WT in the more realistic plasma membrane model, a co-localization of PIP2 and highly unsaturated lipids around the protein was detected. However, the inclusion of a second palmitoylation suppresses these process and prohibits CD44 and FERM binding. Third, the clustering of PIP2 around CD44 allows the CT to stretch and detach from the membrane surface, thus, allowing an easier binding to the FERM. Since these mechanisms critically depend on both the degree of palmitoylations of CD44 and the amount of PIP2 in the membrane, there will be a variety of pathways on controlling the binding of CD44 and FERM, like dimerization of several CD44 units, subsequent palmitoylations of CD44 via PAT, and depalmitoylayions with corresponding enzymes. Besides, PIP2 clustering recruited by membrane proteins is often stable in CG simulations [47,48]. The phenomenon is in agreement with experimental results that demonstrate even small concentrations of PIP2 can greatly enhance the adsorption of FERM [19]. The membrane type and the localization within the cell is of crucial importance as well since raft domains cover typically only a small amount of the membrane surface and PIP2 lipids are especially enriched in certain cellular junctions like lamellipodia of migrating cells. Due to the complexity of the binding mechanism, there are thus several different pathways with which the binding of CD44 and the membrane adaptor can be regulated.

Concluding, we explored the dynamical details of cell adhesion of the protein CD44 translocation in a phase segregated membrane, regulated by palmitoylations and PIP2 lipids and revealed the competitive relationship of acylation modifications on the one hand and lipid-mediated regulation on the other hand at near-to-atomistic resolution. The information of the membrane translocation, structural transformation of CD44 and the binding to membrane adaptors, mediated by PIP2 and different palmitoylations can significantly enhance our comprehension on membrane protein regulation pathways.

## Methods

### Membrane setup

All the simulations conducted for this study were based on Martini force field 2.2 version [49–52]. A lipid bilayer model composed of 40% DPPC / 40% DLiPC / 20% CHOL, that mimic a *Lo/Ld* phase separated membrane, was built by using the script of *insane*.*py* [53] (Fig 1B, Table 1). Thereby, the recently optimized parameters for cholesterol were used [54]. For the systems that contain PIP2 lipids in the lower leaflet, a bilayer model with a composition of 39% DPPC / 39% DLiPC / 20% CHOL / 2% PIP2 in the lower leaflet was constructed, with the upper leaflet left unchanged. PIP2 was modeled with the *sn-1* chain fully saturated, and the *sn-2* chain poly-unsaturated, modeling C16:0-C18:4 PIP2(4, 5). More details of PIP2 parameters [55] are provided in the supporting information (Table A). In our simulations, the PIP2 carries a -5 negative charge. In some control simulations, PIP2 was replaced by dilinoleoyl-phosphatidylserine (DLiPS), using a mole fraction of 10% to keep the same overall charge density. The plasma membrane (PM) model, consisting of 18 lipid species with different degrees of saturation as well as different distributions between the two leaflets, as specified in Table 2. The system was solvated with standard martini water, without utilizing anti-freeze beads. This setup was used in the past for similar system and did not display problems with respect to a potential freezing of the solvent [25].

**Table 1:** An overview of the phase separated membrane models used in the study.

Protein series		CD44-WT	Pal-286	Pal-295	Pal-dual	CD44-WT	Pal-295	Pal-dual	Pal-dual/Depal + FERM	CD44-WT + FERM		Pal-295
Upper leaflet	Lipids	40%DPPC/40%DIiPC/20%CHOL										
	Number	270/270/135										
Lower leaflet	Lipids	40%DPPC/40%DIiPC /20%CHOL				39%DPPC/39%DIiPC/ 20%CHOL/2%PIP2						35%DPPC/35%DIiPC/ 20%CHOL/10%DLiPS
	Number	270/270/135				263/263/135/13						236/236/135/65
**W**		17707				27948			27887			27948
**Ions**		6 (CL-)				6 (CL-)			61 (Na+) 6 (CL-)			6 (CL-)
**Time (μs)**		6.0				6.0			6.0/3.0	3.0	6.0	6.0
**Box Size**		20×20×10 nm^3^				20×20×13 nm^3^						

**Table 2:** An overview of the lipid compositions in the PM membrane model (46×46×10 nm^3^).

**W** (104389)	**Salt** 0.15 mM Cl- (1160) Na+ (2183)	Upper leaflet (3481)	Lipids	**POPX**	**PIPX**	**POPE**	**PAPE**	**DAPE**	**DBSM**	**BNSM**	**DBG1**
				348	895	45	115	35	281	388	89
			Amount	**DBG3**	**DBCE**	**OPC**	**PIDG**	**CHOL**			
				89	29	44	28	1089			
		Lower leaflet (3257)	Lipids	**POPC**	**PIPC**	**POPE**	**PAPE**	**DAPE**	**DBSM**	**BNSM**	**POPS**
				158	406	190	489	149	127	175	69
			Amount	**PAPS**	**PIP2**	**PIPA**	**POP2**	**DBCE**	**PIDG**	**CHOL**	
				279	149	49	50	16	18	927	

The membrane model was built based on the neuronal membrane model developed by Ingólfsson *et al* [36]. All the lipid topologies can be found on our website of *cgmartini*.*nl*. The fully saturated lipids include: DBSM, DBG1, DBG3, DBCE; the mono-unsaturated lipids: POPX, POPE, BNSM, OPC, POPC, POPS, POP2; di-unsaturated lipids: PIPX, PIDG, PIPC, PIPA, PIP2; the highly unsaturated lipid (no less than four double bonds): PAPS, PAPE, DAPE.

### Protein setup

The model for CD44 used throughout the article only consists of the TMD and a shortened CT domain. The CT domain was truncated after 19 residues (Fig 1A), as these have been identified to be responsible for interaction with the FERM domain [56]. The molecular structure of the molecule was constructed with the software Pymol [57] and eventually transferred into the CG structure with the *martinize* tool. The transmembrane domain of CD44 was defined as α-helix, and the cytoplasmic domain was defined according to the structure information when it binds to FERM (PDB: 2ZPY). No further elastic network was applied to the structure of molecule. The parameters of the palmitoylation on the cysteine residues were identical to those in our previous study [33]. The atomistic structure of Radixin-FERM was taken from the protein data bank (PDB: 2ZPY) and transformed into the CG model with the *martinize* tool. An elastic network (EINeDyn) was applied on FERM to maintain the conformational stability during the simulations [58]. Additionally, the *ScFix* modification was added to FERM model to avoid an unphysical flipping of the beta strand residues to the opposite side [59]. The extra constraints are proven to be necessary for keeping the conformation of FERM stable during CG simulation (S11 Fig). CD44 was inserted parallel to the membrane normal into the membrane, with the α-helical TM domain spanning through the full thickness of the bilayer, thereby exposing the CT domain outside the lower leaflet. For simulations involving the association of CD44 and the FERM domain, the latter was positioned at a distance of 1.0 nm away from the lower membrane surface at the beginning of the simulation. FERM was thereby oriented in such a way that none of the subdomains was facing away from the membrane and that distance between each of the subdomains towards the membrane surface was roughly the same (S12 Fig). The initial distance between the CD44-CT domain and FERM was 2.0 nm to avoid a bias to their interaction due to the system setup. In all cases, the membranes were solvated with water and counter ions were added to neutralize the systems. An overview of all the membrane systems simulated is provided in Table 1 and Table 2. The CG models of the lipids and proteins, as well as the system setup used in this study are shown in Fig 1A and 1B.

### Analysis

To characterize the composition of the microdomains around CD44 in the different membranes, we computed the contact fraction of different lipid types. The contact intensity for estimating interaction between lipids and proteins is determined with the gromacs tool of *g_select* which allows one to calculate the number of molecular (specific lipid species) positions for each frame within a cutoff distance from the referring protein. For the ternary system, we found a cutoff of 0.6 nm was sufficient to determine the local environment. For the plasma membrane model, which is less structured and contains a larger variety of lipids, the cutoff was increased to 2.0 nm. This cutoff value was used based on the position where the enrichment of different lipids around the membrane protein shows largest difference (S13 Fig).

### Simulation details

All simulations were conducted with the software package Gromacs-4.6.6 [60]. The lipids and protein parameters used in this study can be downloaded from http://cgmartini.nl. After an energy minimization of the system for 5000 steps using the steepest descent method, an NPT equilibration for 500 ns was conducted with a semi-isotropic coupling to a pressure bath with 1.0 bar, a compressibility constant of 4.5×10^−5^ bar^-1^ and a time constant of 5.0 ps. A constant temperature of T = 295 K was achieved by coupling the system to a heat bath, thereby using a time constant of 1.0 ps. For both the pressure and temperature coupling, the Berendsen method [61] was used. During the equilibration, the protein backbones were constraint by a harmonic potential with a force constant of 1000 kJ∙mol^-1^∙nm^-2^ in all three dimensions. An elastic network with a force constant of 500 kJ∙mol^-1^∙nm^-2^ was applied to beads that were separated by a distance between 0.5 and 0.9 nm. During the simulation of the PM, a harmonic potential with a force constant of 100 kJ∙mol^-1^∙nm^-2^ on the z-axis was applied on the PO4 beads of parts of POPC and PIPC lipids (renamed by POPX and PIPX) to avoid large undulations. Both the electrostatic and Lennard-Jones (LJ) interaction were shifted to zero when they exceeded a cut-off distance of 1.2 nm. Periodic boundary conditions were used for all systems. A time step of 20 fs was used for the numerical integration, and each simulation ran for a duration of 6.0 μs (with the exception of some simulations involving CD44-Pal-dual and FERM, see Table 1). All the simulation snapshots in this study were made by the VMD software package [62].

## Supporting information

S1 Data Excel spreadsheet containing, in separate sheets, the underlying numerical data and statistical analysis for Figure panels 2a, 2b, 2c, 2d, 3d, 3a, 3b, 4a, 4b, 4c, 4d, 5a, 5b, 5d, 6c and 8b. (XLSX)

S1 Text Improved construction and the CG structural details of PIP2 CG model. (PDF)

S1 Fig Number of contacts between CD44 and the lipids of DPPC (black line) and DLiPC (red line).
(TIF)

S2 Fig The DPPC contact intensity along the residues on CD44 of the WT, Pal-286, Pal-295, and Pal-dual. The high DPPC-contact regions are actually the palmitoylation sites which are marked by the dash-line circles.
(TIF)

S3 Fig (A) Membrane contact intensity distribution of the residues on FERM. (B) A 2-D density map showing the position relevance of the FERM domain and the *Ld* phase. (C) CD44 prefers to *Ld* domain when interacting with FERM.
(TIF)

S4 Fig (A-B) Initial and final snapshots of two simulations respectively in which the distance between the FERM subdomains and the lower surface of the bilayer is increased to 2 nm. The color code is consistent with Fig 7 in the main text. (C) Time evolution of the z-position of the three subdomains of FERM relative to the membrane corresponding to the two simulations. The distances were measured from the center of mass of the respective subdomain to the center of the membrane. (D) Lipid contact distribution on the residues calculating from the 0–3 μs simulation timescale, and (E) shows the second simulation starting from a bottom-up rotation of FERM. “Backbone” on protein and “PO4”, “P4”, “P5” beads on lipid head groups were selected for calculating the contact events.
(TIF)

S5 Fig (A) PIP2 molecules mainly distribute in the *Ld* phase. (B) Density distributions of DPPC, DLiPC and PIP2 along the direction perpendicular to the bilayer phase interface.
(TIF)

S6 Fig (A) Snapshot of theCD44-FERM conformation and association on the membrane surface in the presence of PIP2. (B) Interface details of the CD44-FERM complex. (C-D) Evolution of the distances between the residue pairs Q297-D252, K298-N251, K299-F250 and K300-S249 of CD44 and FERM, respectively, for two replicate simulations.
(TIF)

S7 Fig A comparison of radial distribution function (RDF) of PIP2 relative to CD44. The blue line indicates the case of adding FERM.
(TIF)

S8 Fig Distance revolutions between the CD44-Pal-dual and FERM (black and gray lines), and the cases of the depalmitoylated-CD44 and FERM (red and purple lines). The starting time began from the end of simulation (3.0 μs) of CD44-Pal-dual/FERM in Fig 8B.
(TIF)

S9 Fig (A) Distance between CD44 and FERM as a function of the simulation time. The snapshots correspond to the initial protein-separated state and final protein-associated states. (B) Lateral mean square deviation (MSD) of FERM on the membrane during the 0–6 μs simulation.
(TIF)

S10 Fig (A) Presentations of CD44 homodimer forming in the membrane model. The backbones of the two CD44 monomers are distinguished in blue and red respectively. The cysteine residues are displayed in yellow. A snapshot in a lateral view is provided to demonstrate the different positions of Cys-286 and Cys-295 on CD44 dimer. PIP2 lipids surrounding CD44 dimer are highlighted in ochre. (B) Residue contact matrix in CD44 dimerization. Only the TM domains are calculated as donated by Residue Index1 and 2; (C) Distance evolutions of the inter-Cys286 and the inter-Cys295.
(TIF)

S11 Fig (A) The initial (red) and final (green) structures of FERM obtained from the all-atom simulation. (B)Time evolutions of the secondary structures of FERM during the 100 ns all-atom simulation. (C) A comparison of RMSFs of FERM Cα atoms calculated within the 100 ns all-atom and CG simulations. **Note:** The force field of Gromacs-53a6 is used to simulate the protein in a system with box size of 8×8×8 nm^3^. The protein structure was obtained from the protein data bank (PDB code: 2ZPY) and transferred into a MD model with the build-in tool *pdb2gmx* of gromacs. SPC water model was used to solvate the protein and counter ions were added for neutrality of the net charge. Further simulation parameters were chosen in analogy to our previous study (*J*. *Chem*. *Inf*. *Model*. 2017, 57, 1375−1387). In order to produce RMSF of CG model in a comparable condition with the all-atom model, FERM (with an elastic network and ScFix) was just solvated by standard CG water and ions and ran for 100 ns.
(TIF)

S12 Fig Snapshot of the initial state of the CD44/FERM system in ternary lipid bilayer. (TIF)

S13 Fig RDFs of (A) PIP2, (B) Saturated lipids and (C) unsaturated lipids around CD44-WT or CD44-Pal-dual, respectively. The results show that, within distance of 2.0 nm, the enrichment of lipid groups can be distinguished between the WT and Pal-dual. On this basis, the cutoff distance for calculating the lipid contact was set as 2.0 nm.
(TIF)
