# Supplementary material for: Molecular mechanism for bidirectional regulation of CD44 for lipid raft affiliation by palmitoylations and PIP2
Source: PLoS Comput Biol. 2020 Apr 9;16(4):e1007777. doi: 10.1371/journal.pcbi.1007777 (PMC7173942; doi:10.1371/journal.pcbi.1007777)
Supplement: S1 Text — (PDF) [file pcbi.1007777.s002.pdf]

COARSE GRAINING OF PIP-4,5 [POP6]

The parameters for PIP-4,5 used in this study were obtained from newly-parameterized Martini phosphatidylinositol phosphates (unpublished). These bonded parameters were derived using a bottom-up approach of PIPs embedded in POPC bilayers in the GROMOS53a6 united-atom force-field (Oostenbrink et al., 2004), and posteriorly validated in coarse-grained membranes against a set of experimental properties. To name a few: bilayer thickness, area per lipid, z-position of the phosphate linker (i.e. the z-component of the average position of PO4 respect to the bilayer plane), tilt angle (angle between the vector connecting C1 and the center of mass of C2 and C3, and the bilayer normal), rotation angle (angle between the vector connecting C2 and C3, and the bilayer normal), and the ability of POP6 to form clusters in presence of Ca^{2+} (Li et al., 2009; Wu et al., 2014; Slochower et al., 2013; Ingólfsson et al., 2014).

Table A. Coarse-grained model and parameters of PIP2. The bead order and type (by circles with different colors) is shown in the left figure. The topology is derived from the model developed by Lopez et al. with these modifications: 1. The beads of the head ring structure are all defined as "P1"; 2. The bead type on the *sn-2* hydrocarbon chain is changed to "C4" to represent the unsaturated carbon bonds; 3. New angles and dihedral angles are enriched in the topology to refine PIP2 model. More detail like the bond length can be seen in the martini_v2.0_PIP2.itp file. (P4 and P4 are both attached to C2 and C3, they differ in their bonded distances and orientation respect to the plane of the inositol ring; P5 is planar respect to the ring and P4 is slightly tilted to the front, which makes P5 lie farther away from the membrane towards the aqueous environment. Angle C2-C1-PO4 is used to adjust the head-tail angle).

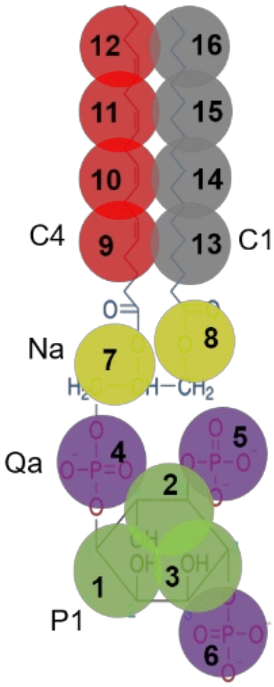

**Angle**	Bead orders	Function	Angle (°)	Force (kJ/mol)
Head group	1-4-7	2	140	25
	4-7-8	2	120	25
	4-7-9	2	180	25
	2-1-4 (New)	2	140	400
Tail-*Sn-1*	8-13-14	2	180	25
	13-14-15	2	180	25
	14-15-16	2	180	25
Tail-*Sn-2*	7-9-10	2	100	10
	9-10-11 (Modified)	2	120	45
	10-11-12 (Modified)	2	180	25
**Dihedral angle**	3-1-4-7	1	-30	5
	5-1-2-3 (New)	1	50	12
	6-2-1-3 (New)	1	180	100
