# Supplementary figures and images for: Molecular mechanism for bidirectional regulation of CD44 for lipid raft affiliation by palmitoylations and PIP2
Source: PLoS Comput Biol. 2020 Apr 9;16(4):e1007777. doi: 10.1371/journal.pcbi.1007777 (PMC7173942; doi:10.1371/journal.pcbi.1007777)

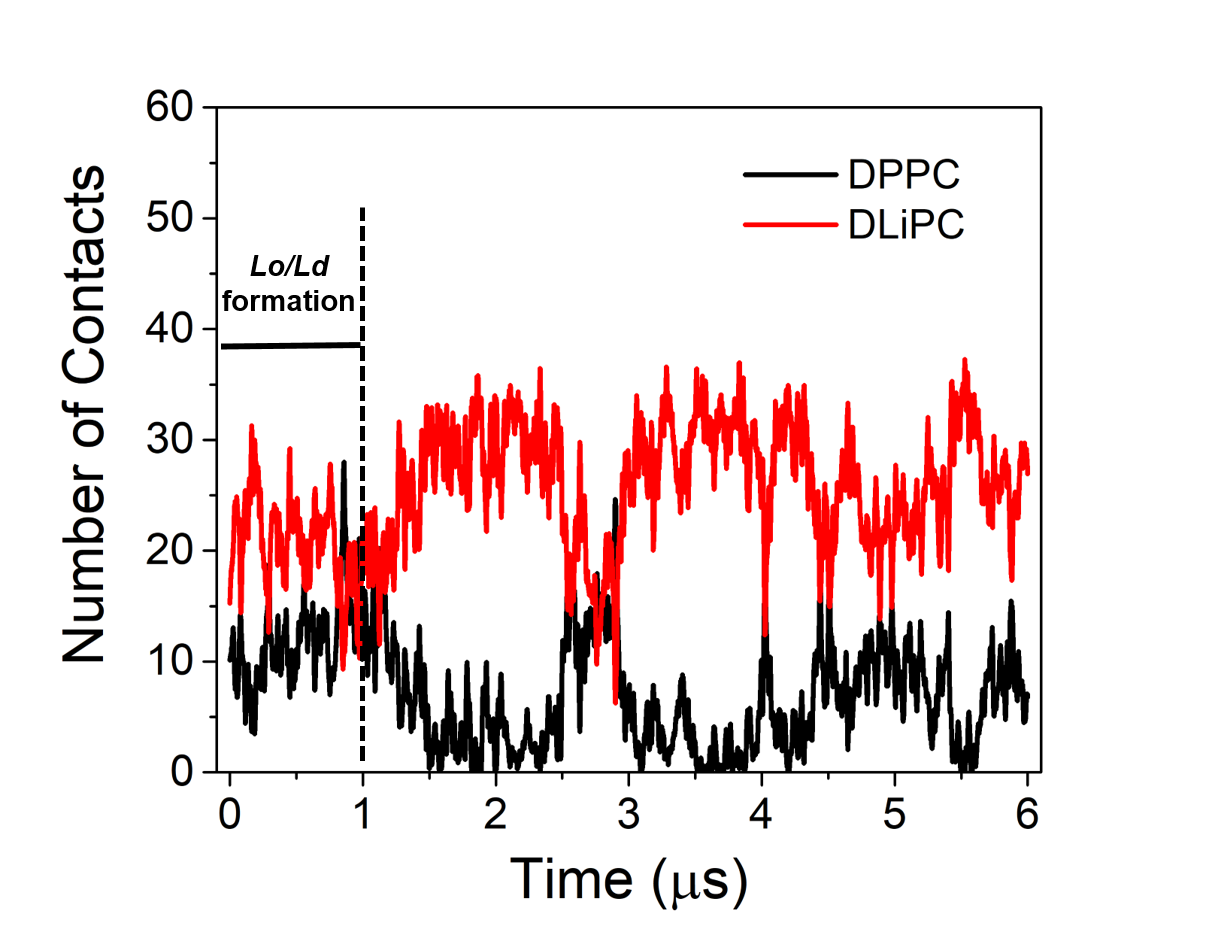

Supplement: S1 Fig — Number of contacts between CD44 and the lipids of DPPC (black line) and DLiPC (red line). (TIF) [file pcbi.1007777.s003.tif]

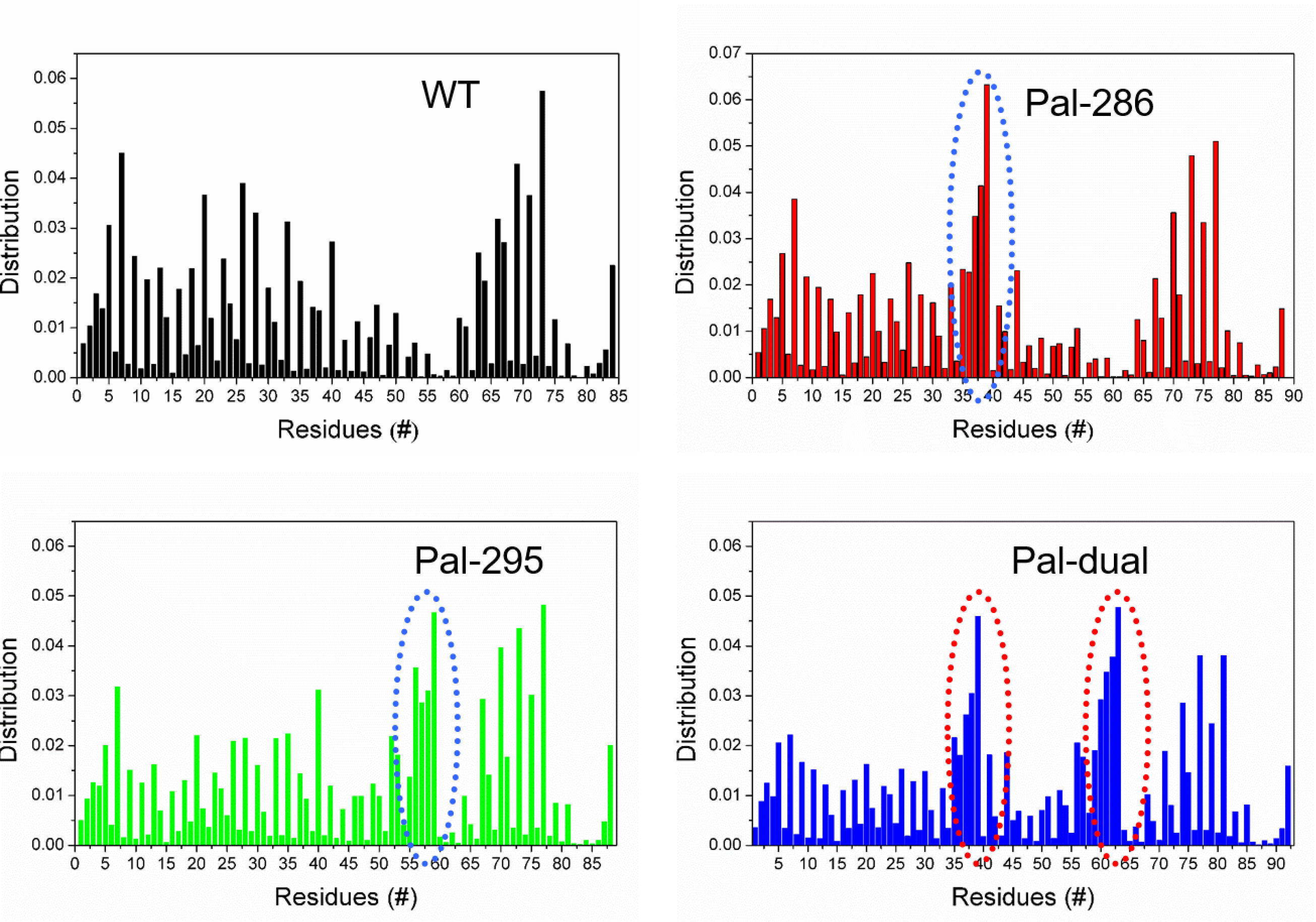

Supplement: S2 Fig — The high DPPC-contact regions are actually the palmitoylation sites which are marked by the dash-line circles. (TIF) [file pcbi.1007777.s004.tif]

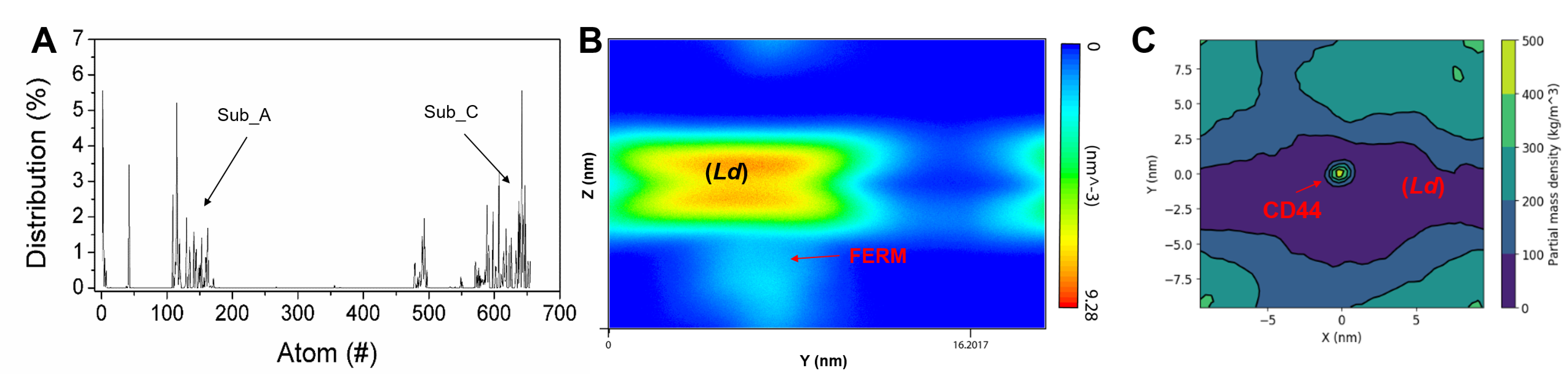

Supplement: S3 Fig — (A) Membrane contact intensity distribution of the residues on FERM. (B) A 2-D density map showing the position relevance of the FERM domain and the Ld phase. (C) CD44 prefers to Ld domain when interacting with FERM. (TIF) [file pcbi.1007777.s005.tif]

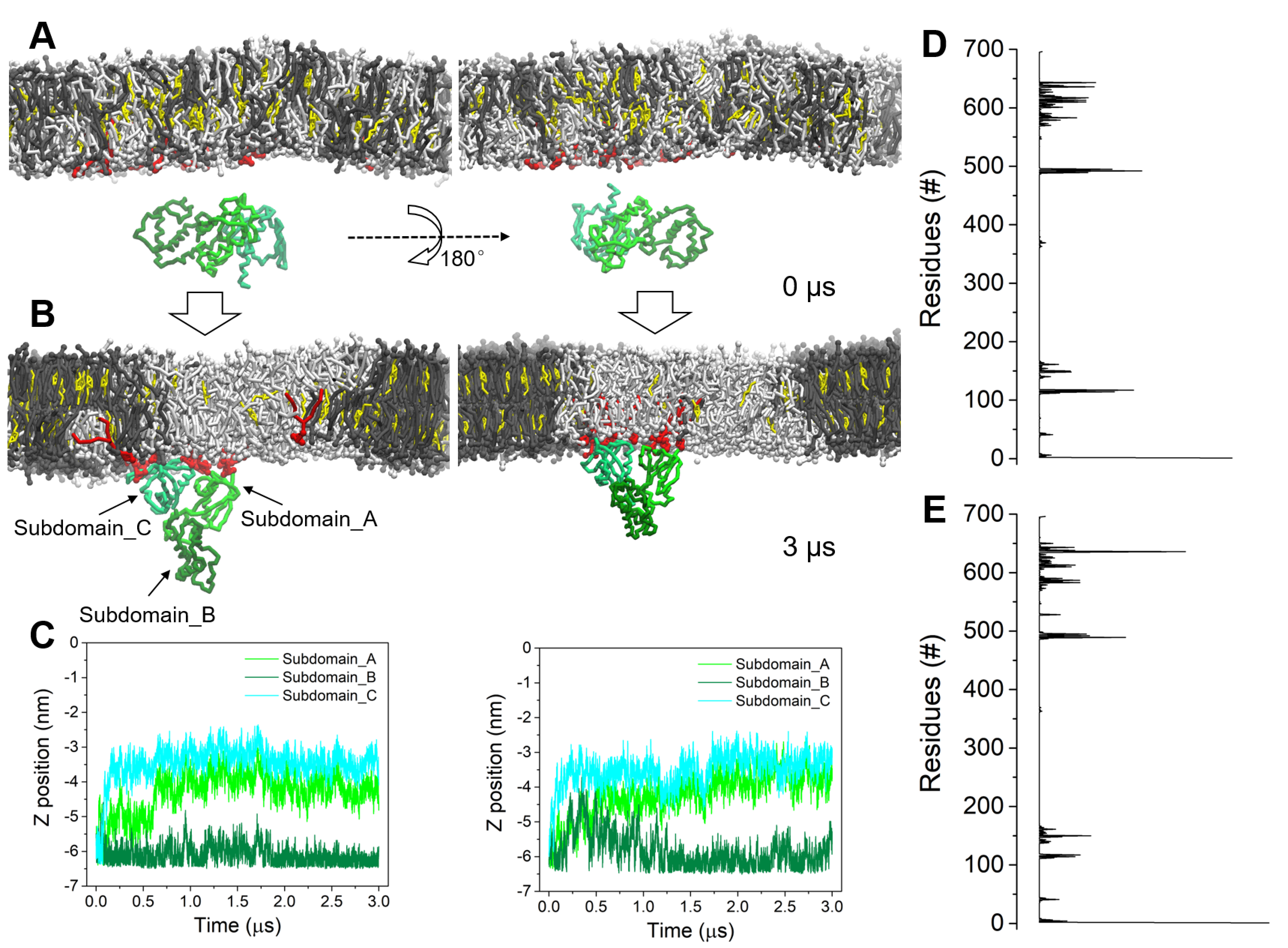

Supplement: S4 Fig — (A-B) Initial and final snapshots of two simulations respectively in which the distance between the FERM subdomains and the lower surface of the bilayer is increased to 2 nm. The color code is consistent with Fig 7 in the main text. (C) Time evolution of the z-position of the three subdomains of FERM relative to the membrane corresponding to the two simulations. The distances were measured from the center of mass of the respective subdomain to the center of the membrane. (D) Lipid contact distribution on the residues calculating from the 0–3 μs simulation timescale, and (E) shows the second simulation starting from a bottom-up rotation of FERM. “Backbone” on protein and “PO4”, “P4”, “P5” beads on lipid head groups were selected for calculating the contact events. (TIF) [file pcbi.1007777.s006.tif]

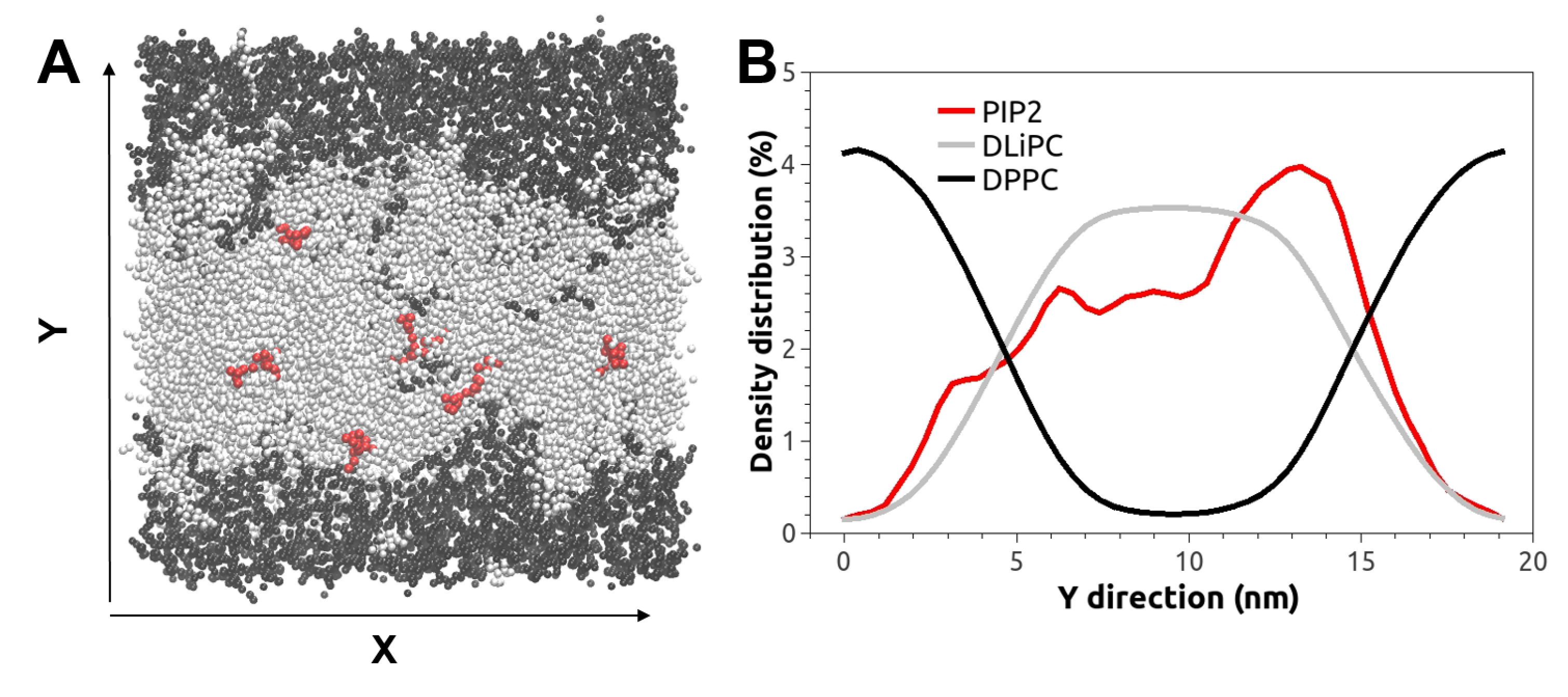

Supplement: S5 Fig — (A) PIP2 molecules mainly distribute in the Ld phase. (B) Density distributions of DPPC, DLiPC and PIP2 along the direction perpendicular to the bilayer phase interface. (TIF) [file pcbi.1007777.s007.tif]

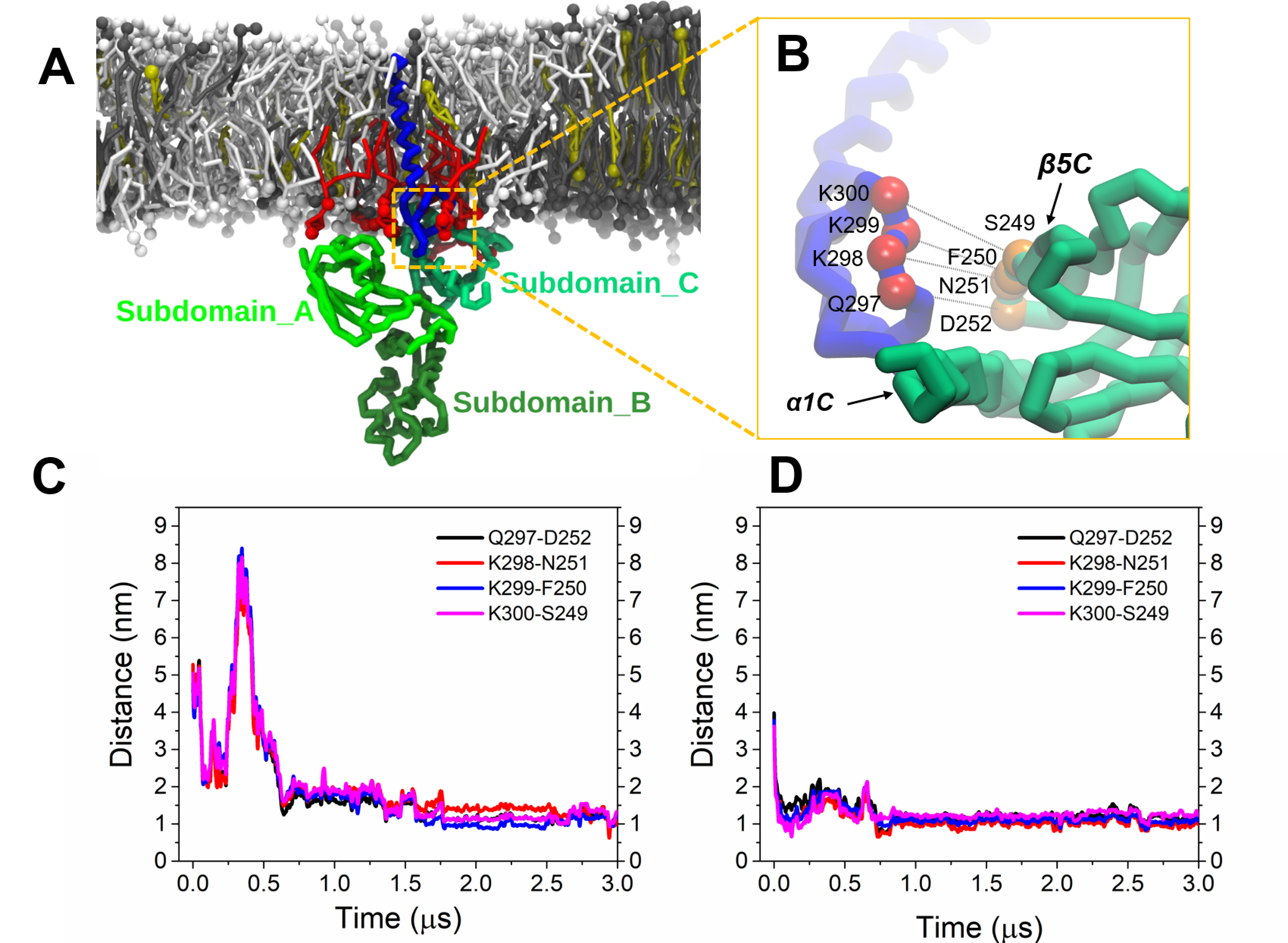

Supplement: S6 Fig — (A) Snapshot of theCD44-FERM conformation and association on the membrane surface in the presence of PIP2. (B) Interface details of the CD44-FERM complex. (C-D) Evolution of the distances between the residue pairs Q297-D252, K298-N251, K299-F250 and K300-S249 of CD44 and FERM, respectively, for two replicate simulations. (TIF) [file pcbi.1007777.s008.tif]

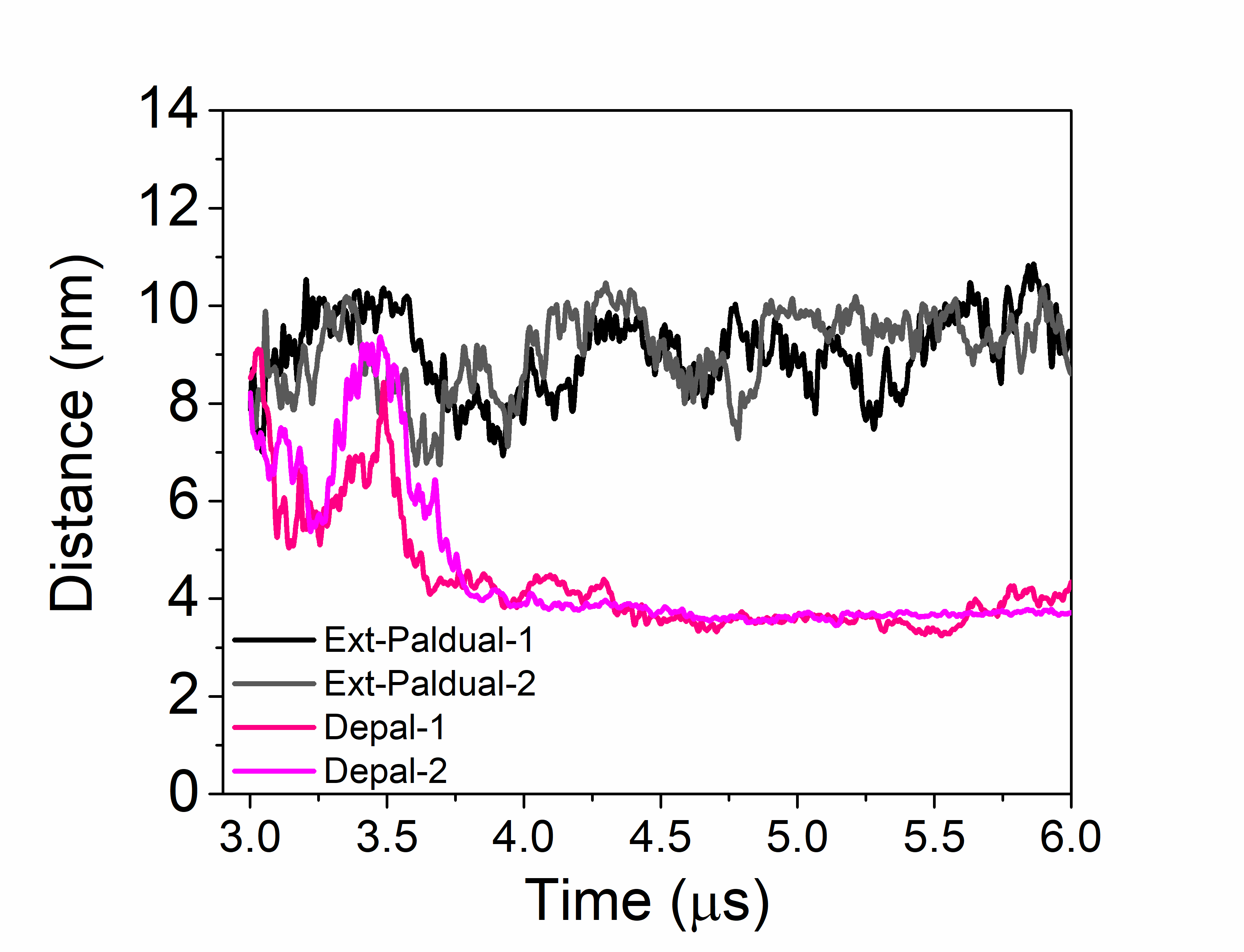

Supplement: S8 Fig — Distance revolutions between the CD44-Pal-dual and FERM (black and gray lines), and the cases of the depalmitoylated-CD44 and FERM (red and purple lines). The starting time began from the end of simulation (3.0 μs) of CD44-Pal-dual/FERM in Fig 8B. (TIF) [file pcbi.1007777.s010.tif]

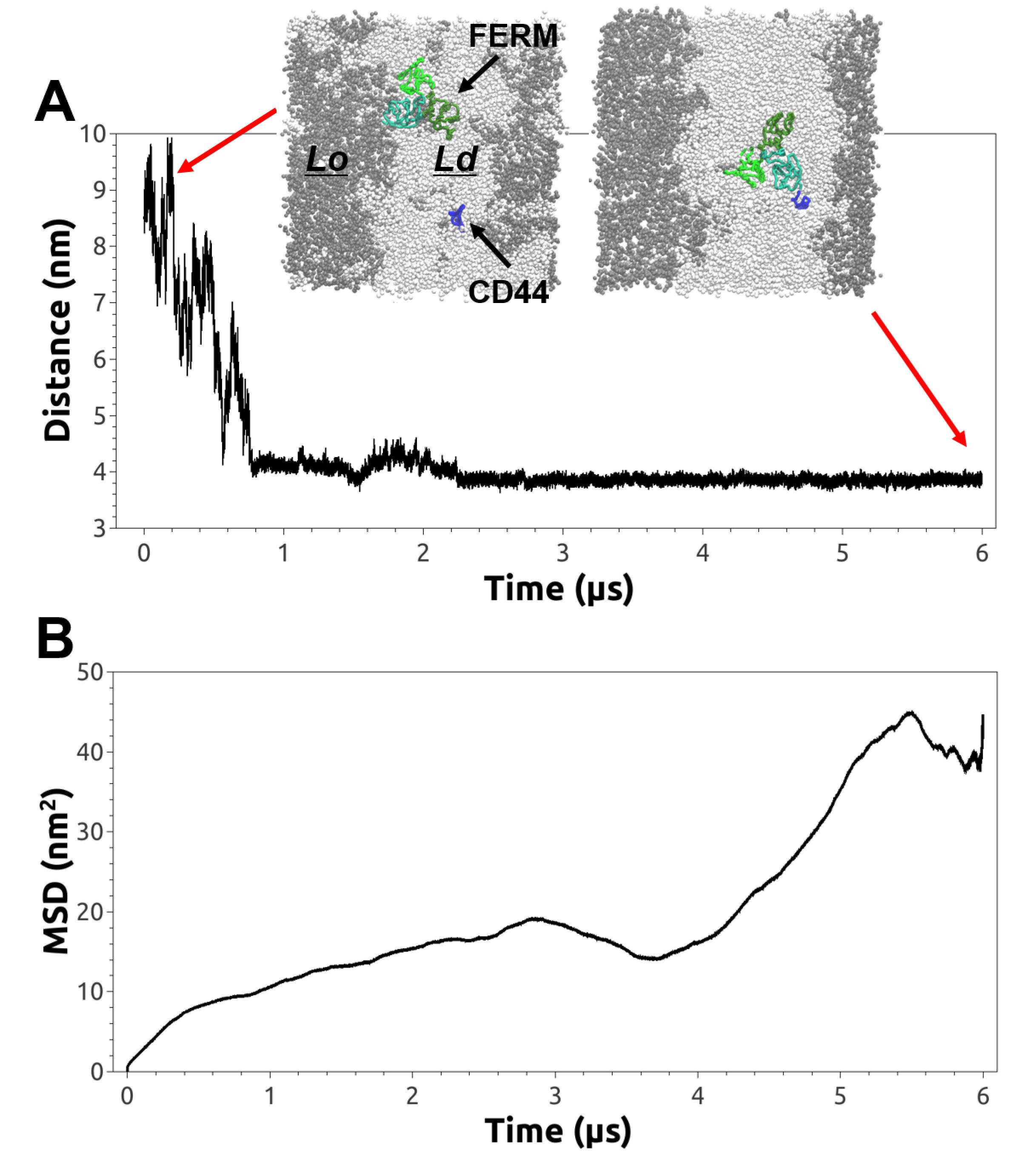

Supplement: S9 Fig — (A) Distance between CD44 and FERM as a function of the simulation time. The snapshots correspond to the initial protein-separated state and final protein-associated states. (B) Lateral mean square deviation (MSD) of FERM on the membrane during the 0–6 μs simulation. (TIF) [file pcbi.1007777.s011.tif]

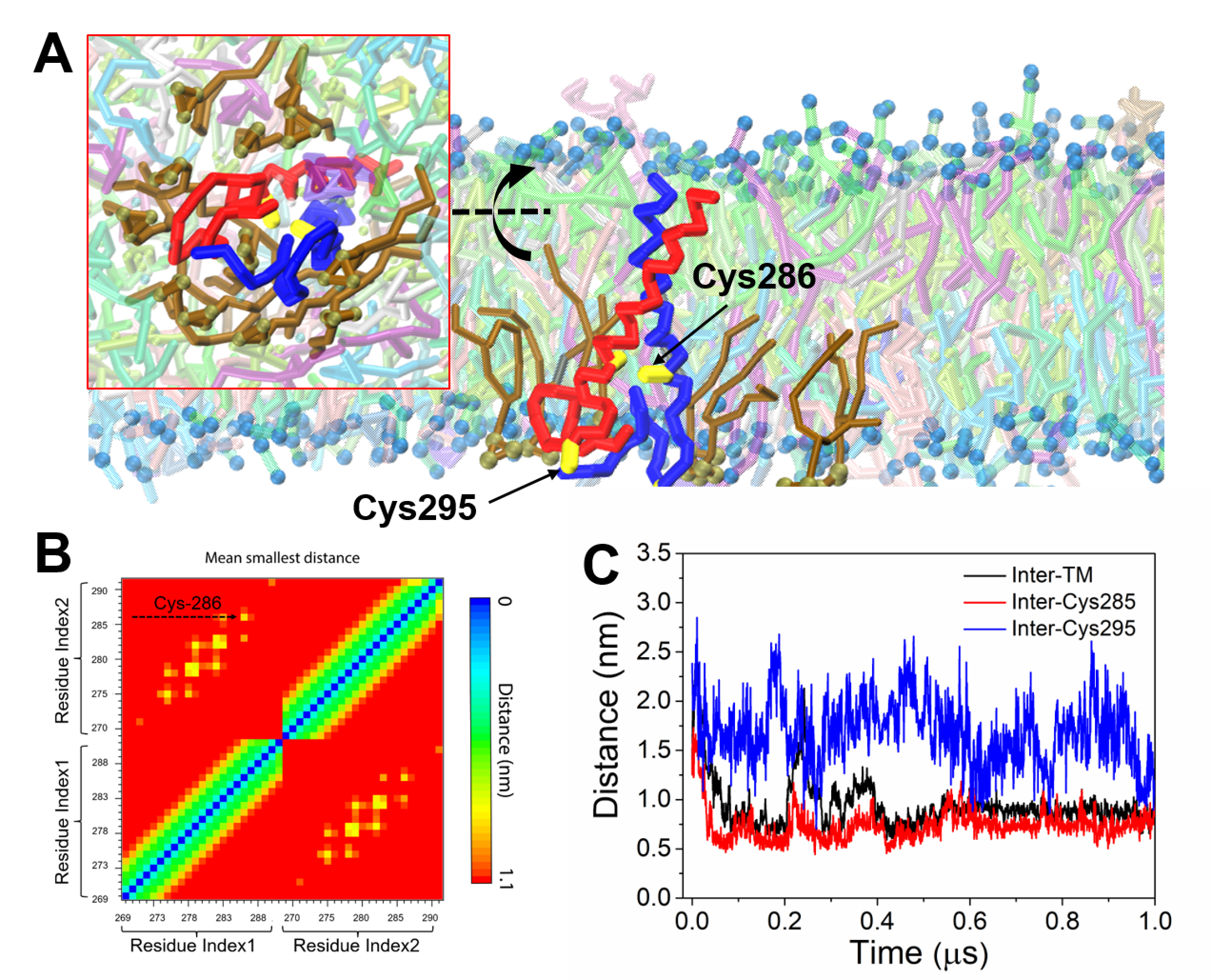

Supplement: S10 Fig — (A) Presentations of CD44 homodimer forming in the membrane model. The backbones of the two CD44 monomers are distinguished in blue and red respectively. The cysteine residues are displayed in yellow. A snapshot in a lateral view is provided to demonstrate the different positions of Cys-286 and Cys-295 on CD44 dimer. PIP2 lipids surrounding CD44 dimer are highlighted in ochre. (B) Residue contact matrix in CD44 dimerization. Only the TM domains are calculated as donated by Residue Index1 and 2; (C) Distance evolutions of the inter-Cys286 and the inter-Cys295. (TIF) [file pcbi.1007777.s012.tif]

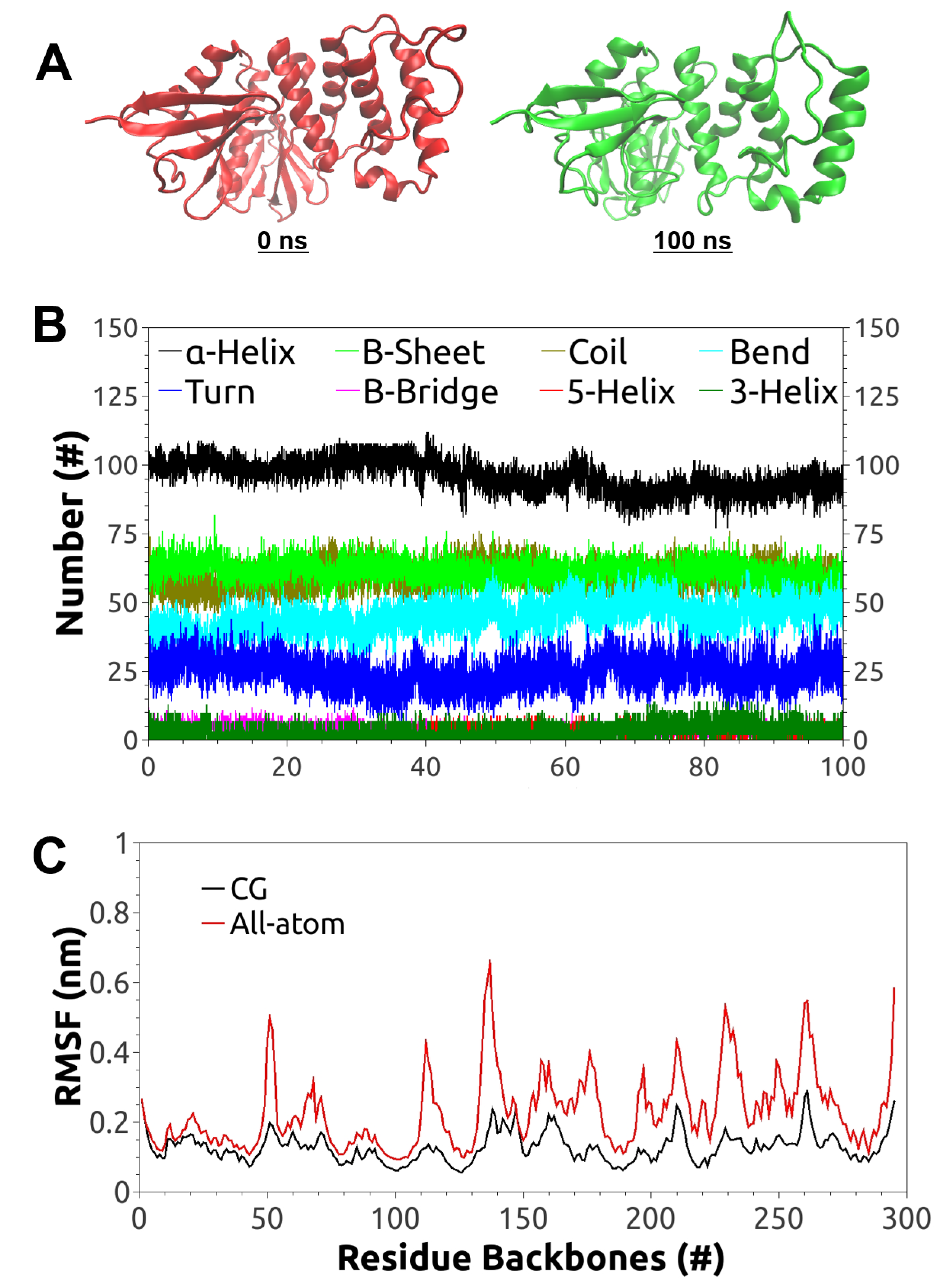

Supplement: S11 Fig — (A) The initial (red) and final (green) structures of FERM obtained from the all-atom simulation. (B)Time evolutions of the secondary structures of FERM during the 100 ns all-atom simulation. (C) A comparison of RMSFs of FERM Cα atoms calculated within the 100 ns all-atom and CG simulations. Note: The force field of Gromacs-53a6 is used to simulate the protein in a system with box size of 8×8×8 nm3. The protein structure was obtained from the protein data bank (PDB code: 2ZPY) and transferred into a MD model with the build-in tool pdb2gmx of gromacs. SPC water model was used to solvate the protein and counter ions were added for neutrality of the net charge. Further simulation parameters were chosen in analogy to our previous study (J. Chem. Inf. Model. 2017, 57, 1375−1387). In order to produce RMSF of CG model in a comparable condition with the all-atom model, FERM (with an elastic network and ScFix) was just solvated by standard CG water and ions and ran for 100 ns. (TIF) [file pcbi.1007777.s013.tif]

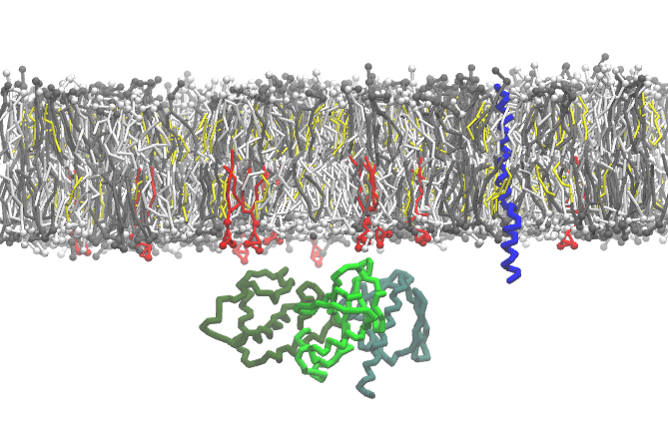

Supplement: S12 Fig — (TIF) [file pcbi.1007777.s014.tif]

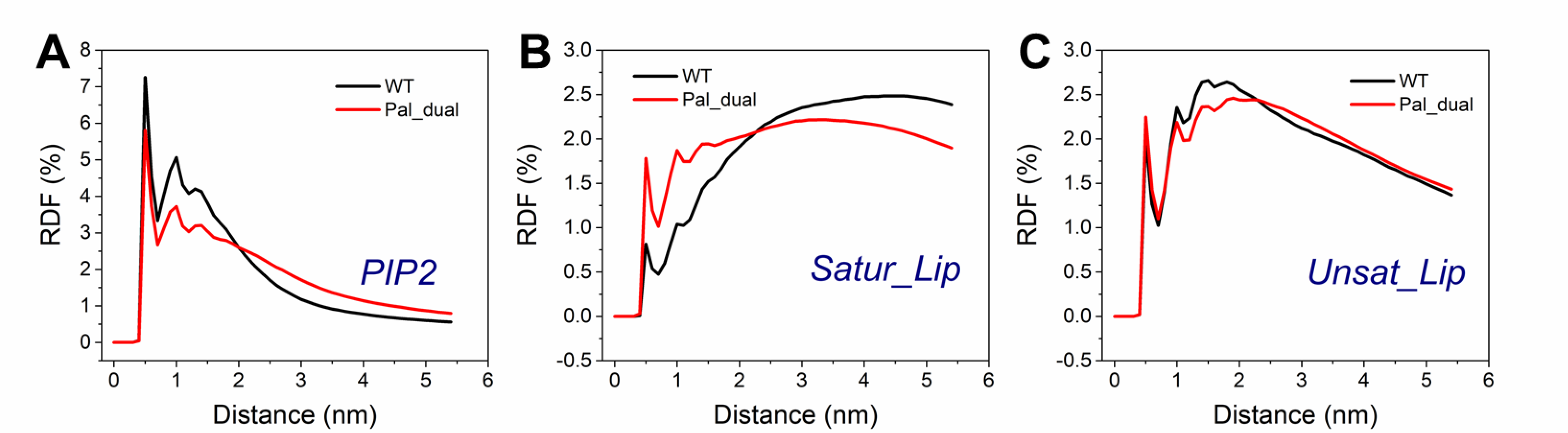

Supplement: S13 Fig — RDFs of (A) PIP2, (B) Saturated lipids and (C) unsaturated lipids around CD44-WT or CD44-Pal-dual, respectively. The results show that, within distance of 2.0 nm, the enrichment of lipid groups can be distinguished between the WT and Pal-dual. On this basis, the cutoff distance for calculating the lipid contact was set as 2.0 nm. (TIF) [file pcbi.1007777.s015.tif]
